# Sensitive Detection of Specific Volatile Organic Compounds by Functionalized Transition Metal Dichalcogenide Monolayers

**DOI:** 10.1021/acs.langmuir.5c02852

**Published:** 2025-08-12

**Authors:** Saba Khan, Tanveer Hussain, Chandra Veer Singh, Nacir Tit

**Affiliations:** † Department of Materials Science and Engineering, 7938 University of Toronto, Toronto, Ontario M5S 3E4, Canada; ‡ School of Science and Technology, 1319 University of New England Armidale, New South Wales 2351, Australia; § Department of Physics, College of Science, 155096 UAE University, P.O. Box 15551, Al-Ain 15551, United Arab Emirates; ∥ National Water and Energy Centre, 155096 UAE University, P.O. Box 15551, Al-Ain 15551, United Arab Emirates

## Abstract

Timely detection of liver cirrhosis (LC) is critical for effective clinical management and improved patient outcomes. Among emerging diagnostic approaches, detection of volatile organic compounds (VOCs), related to LC, offers a noninvasive, rapid, and cost-effective alternative to conventional methods. In this work, we employed spin-polarized density functional theory (DFT) to systematically investigate the interaction of LC-related VOCs using transition-metal dichalcogenides (TMDs), specifically WX_2_ monolayers (X = S, Se_2_). Five VOCs, namely, 2-pentanone, dimethyl sulfide (DMS), isoprene, limonene, and methanol, were selected based on their experimental association with LC. To enhance the sensitivity and selectivity of TMDs, Mn and Fe atoms were used to dope the chalcogen sites of WX_2_, inducing strong dipole moments and improved van der Waals (vdW) interactions. The doped systems demonstrated significantly higher adsorption energies (*E*_ads_, 1.5–2.1 eV), charge transfer (Δ*q* = 0.4–0.8 e), and magnetization changes (Δ*M* ≠ 0) for VOCs compared to air molecules (*E*_ads_ < 0.5 eV, Δ*q* < 0.1 e, Δ*M* = 0), confirming strong selectivity. Work function shifts Δϕ > 0.4 eV (for VOCs) and changes in the density of states near the Fermi level further support enhanced electronic response upon VOC adsorption. Our study offers atomic-scale insights into adsorption energetics, charge transfer, and electronic structure modulation that can guide future experimental efforts in nanobiosensor development. We also critically examine the scope and limitations of our theoretical framework, emphasizing the need for experimental validation to translate these findings into practical diagnostic technologies.

## Graphical abstract

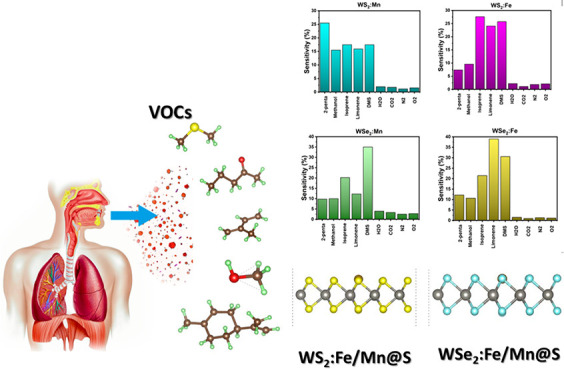

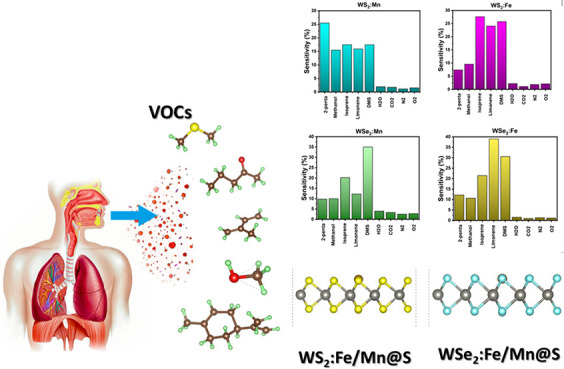

## Introduction

Liver cirrhosis (LC) is a severe chronic liver disease characterized by progressive fibrosis, hepatocellular dysfunction, and the architectural distortion of the liver parenchyma. It is a major global health burden and is ranked among the leading causes of mortality and morbidity worldwide, accounting for over 1.32 million deaths annually.^1^ The main causes of cirrhosis are chronic diseases like alcoholic liver disease, chronic hepatitis (including B, C, and D types), nonalcoholic steatohepatitis (NASH), autoimmune hepatitis, and several genetic disorders.^2^ One of the critical challenges in the management of LC is its asymptomatic early stage, where patients often remain undiagnosed until the disease reaches an irreversible state.^3^ Early detection of LC is crucial for effective intervention, as timely therapeutic measures can delay progression, prevent hepatic decompensation, and significantly improve survival rates.^4^

In recent years, volatile organic compounds (VOCs) in exhaled breath have emerged as promising biomarkers for noninvasive liver disease diagnosis. The idea of using breath analysis for liver disease detection can be traced back to ancient medicine, where physicians noted a distinctive sweet or musty odor in cirrhotic patients, often referred to as “fetor hepaticus”.^5^ From the perspective of VOCs’ analysis, in 1971, Linus Pauling discovered that healthy human breath contains about 200 VOCs.^6^ Hence, the question remains as to which appropriate VOCs to select as LC biomarkers beyond which critical density to decide about the cirrhosis diagnosis and, finally, what materials should be suitable for the detection. Modern analytical techniques have identified specific VOCs that accumulate in the breath of patients with LC due to impaired hepatic metabolism.^7^ The liver plays a critical role in metabolizing endogenous and exogenous compounds, and hepatic dysfunction leads to the accumulation of certain VOCs in the circulation, which are eventually exhaled through the lungs. Several studies have identified 2-pentanone (C_5_H_10_O), dimethyl sulfide (C_2_H_6_S) (DMS), isoprene (C_5_H_8_), limonene (C_10_H_16_), and methanol (CH_3_OH) as key VOCs associated with liver dysfunction.^7,8^

The detection and quantification of VOCs in exhaled breath require highly sensitive analytical techniques such as gas chromatography–mass spectrometry (GC-MS), electronic nose (e-Nose) sensors, and electrochemical sensing methods. GC-MS is the gold standard for VOC analysis, offering high sensitivity and specificity, but it is expensive and requires specialized laboratory equipment.^9,10^ The e-Nose, which uses nanoporous gold particles as sensors, has gained significant attention for its potential to provide real-time, noninvasive breath analysis.^11^ Electrochemical sensors, utilizing materials such as thiol-capped gold nanoparticles, have also demonstrated high sensitivity in detecting LC-related VOCs like limonene.^12^ Recent studies have further integrated machine learning algorithms with breath analysis to enhance diagnostic accuracy, achieving AUROC values above 0.90, indicating a high potential for early stage liver cirrhosis detection.^13^

Transition metal dichalcogenides (TMDs) are a class of layered 2D materials known for their tunable electronic properties, strong spin–orbit coupling, and bandgap variability, making them ideal for applications in electronics, optoelectronics, and gas sensing.^14^ Among them, tungsten-based TMDs (WS_2_ and WSe_2_) exhibit superior structural and electronic stability, with their bandgap transitions and defect-engineered structures enhancing their potential as sensors.^15^ The credits of synthesizing a TMD monolayer (ML) goes back to the year 2010 to the two groups of Splendiani et al.^16^ and Mak et al.,^17^ which succeeded in fabricating MoS_2_ ML and detecting its corresponding embryonic photoluminescence (PL). Such PL was taken as an indication of the crossover from indirect (*E*_g_ = 1.26 eV) to direct bandgap (*E*_g_ = 1.90 eV) transitions along the attempts in growing thinner MLs.^16,17^ The synthesis method was top-down based on the microexfoliation technique like the one used by the graphene inventors. Nevertheless, TMD MLs (MX_2_, M = Mo, W and X = S, Se, Te) have properties distinct from those of the semimetal graphene by possessing a direct bandgap and can be used in electronics as transistors and in optics as emitters and detectors.^18–21^ On large scale, bottom-up growth techniques are broadly used for the growth of TMD MLs including chemical vapor-phase deposition (CVD),^22^ molecular beam epitaxy (MBE),^23^ and atomic layer deposition (ALD).^24^ Artificial intelligence (AI) has recently been explored in designing 2D TMD materials for specific bandgap engineering applications such as memory technology and neuromorphic computing.^25^ Novel Janus TMD MLs, such as MoSSe and WSSe, appeared with fascinating properties and extended the domain of applications such as nanoelectronics, optoelectronics, valleytronics, and catalysis.^26,27^

In the gas sensing field, sensors based on metal oxides (e.g., SnO_2_ and ZnO) have limitations to become optimized at high temperatures and limited sensitivity on the order of parts per million (ppm).^28,29^ For biomedical applications, such as detecting VOCs related to cancer diseases, sensors with higher responses at the scale of parts per billion (ppb) and operating at room temperature (RT) are needed.^30–36^ In the case of the chemisorption type of interaction between VOCs and adsorbent, selectivity toward VOC detections were reported, such as on pristine B_3_C_2_N_3_,^30^ B_2_N,^31^ pentagonal B_2_C,^32^ and B_3_O_3_ ^33^ monolayers. However, the biosensors exhibited a very long recovery time and consequently were disposable. To secure a reusable biosensor working with high sensitivity and under ambient conditions, the physisorption type of interaction should be explored. Such regulated interaction can be achieved, for instance, using functionalized TMDs and MXenes.^34–40^ Recently, Alfalasi et al.^34^ have shown that the doping with transition metal (TM, such as Mo, Fe, Co, Cu) atom when targeting the chalcogenide site can induce both magnetic and electric dipole moments into the TMD (MoS_2_) ML. Enhancing the polarity of the TMD monolayer can induce electric dipole moments in many localities within the VOC molecules, and consequently, stronger van der Waals (vdW) interactions get triggered. Thus, physisorption interactions with the VOCs (e.g., lung cancer biomarkers) were much greater than those with interfering air molecules. Kumar et al.^35^ explored titanium carbide MXenes to report an efficient detection of VOCs related to Alzheimer’s disease (AD) biomarkers. Chen et al.^41^ proposed a TMDs/MXene hybrid sensor based on a Ti_3_C_2_T_*x*_/WSe_2_ bilayer. This latter sensor was reported to possess a sensitivity of about 12-fold in comparison to that of pristine Ti_3_C_2_T_*x*_ MXene. Besides, the hybrid sensor exhibited low noise level and ultrafast response/recovery time and consequently is reusable.^41^

The present work aims to explore the TM-doped TMDs (WX_2_, with X = S, Se) for the efficient detection of selected VOCs as LC biomarkers. We have focused on five VOCs, which have proven to be indicative of LC. We have doped WS_2_ and WSe_2_ with selected TM atoms, for example, Fe and Mn at chalcogenide S/Se sites (WS_2_:TM@S and WSe_2_:TM@Se) to assess their ability as efficient sensors toward the proposed VOCs. We emphasize that the substitute TM doping should target the chalcogenide (X) site to enhance the adsorption energies with VOCs and enhance their selective detection, as was demonstrated in our recent work.^37^ The paper is organized as follows: the next section presents the details of the computational methods used for the simulations. The third section presents an elaborate analysis and explanation of the results. The final section summarizes our main findings and conclusions.

## Computational Section

In this work, we have utilized state-of-art techniques, based upon Density Functional Theory (DFT) to study the ground state properties (i.e., adsorption energy, atomic structure, electronic, and magnetic properties) as implemented in the Vienna Ab-initio Simulation Package (VASP).^42^ VASP is based upon the projected plane wave as a basis set. In the context of VASP, pseudopotentials are used to describe the ionic potential, while the valence-electron wave function is expressed in a plane-wave expansion within an energy cutoff (*E*_cut_) of 520 eV. The exchange-correlation functional is approximated by employing the general gradient approximation (GGA) of Perdew–Burke–Ernzerhof (PBE).^43^ The Monkhorst–Pack technique is used for the sampling of the Brillouin zone.^44^ Based on our computational supercell having the size of 5 × 5 primitive cells (PCs), we used a k-mesh grid of 5 × 5 × 1 for atomic relaxation and a denser grid of 11 × 11 × 1 in calculating the electronic density of states (DOS). Note that the DFT-D3 scheme of Grimme was applied to take account of the van der Waals (vdW) interactions between the VOC molecules and the substrate.^45^ For the convergence criteria of the total energy of the systems and atomic force, we used tolerances of 10^–6^ eV and 0.01 eV/Å, respectively. The width of the Gaussian smearing in the statistics of the DOS calculations of 0.05 eV was used. The charge exchanges between molecules and TMD monolayers are calculated using the Bader charge analysis.^46^

To study the adsorption, the VOCs and interfering air molecules were individually introduced on the surface of TM-doped TMDs within a distance of ∼2.0 Å, and atomic relaxations were performed. Initial configurations of molecules on different sites and in different orientations were tested. Global minimums were obtained and found to correspond to the configurations of molecules aligned parallel to the substrate. The results of the total energy calculations are explored to calculate the molecular adsorption energy, which is defined by

$$E_{ads}=E_{\left(TMD+VOC\right)}-E_{TMD}-E_{VOC}$$ 1

where *E*_(TMD+VOC)_, *E*_TMD_, and *E*_VOC_ are the total energies of the TMD monolayers with and without VOC and isolated VOC, respectively.

To decide whether the sensor should be reusable or disposable, one may estimate the recovery time^47^ given by

$$\tau =\nu_{o}\;\exp\left[-\frac{E_{ads}}{k_{B}T}\right]$$ 2

where *ν*_o_ = 10^13^ s^–1^ is the attempt frequency,^48^ *E*_ads_ is the adsorption energy, and *k*_B_ and *T* are the Boltzmann constant and the absolute temperature, respectively. In the present case, the sensing material involves TMD doped with transition metal atoms. Hence, in all of our calculations, we included spin polarization. Meanwhile, it is worth emphasizing that all of the adsorption cases (five VOCs and four interfering air molecules) exhibit physisorption processes. The results of atomic relaxations, adsorption energy, spin-polarized DOS and bands, charge transfer, and sensor response are discussed in the next section.

## Results and Discussion

### Pristine WX_2_ (X = S, Se)

Figure 1 displays the relaxed atomic structures of pristine and Mn/Fe-doped TMDs (WX_2_, with X = S and Se) using a computational supercell of size 5 × 5 primitive cells (PCs). The structure belongs to a triangular 2D Bravais lattice with three atoms/PC, under space group *P*6_3_/*mmc* #194.^49,50^ As mentioned in the introduction, the doping targeted the anion site, as was proven to yield a strong electric dipole moment effective enough to trigger strong vdW interactions between the VOCs and the substrate. Figure 1a–c shows the atomic structures of WS_2_, WS_2_:Mn@S, and WS_2_:Fe@S in both top and side views, whereas Figure 1d–f displays the other structures of WSe_2_, WSe_2_:Mn@Se, and WSe_2_:Fe@Se. The geometric parameters are also shown. For the pristine WS_2_ monolayer (ML), the calculated lattice constant and bond length are *a* = 3.157 Å and *b* = 2.414 Å, which compare favorably with the experimental data *a* = 3.153 Å and *b* = 2.405 Å reported by Gutierrez et al.^49^ Whereas for pristine WSe_2_ ML, our results are *a* = 3.250 Å and *b* = 2.564 Å in good agreement with the experimental data *a* = 3.247 Å and *b* = 2.555 Å reported by Cai and co-workers.^50^

**Figure 1:**
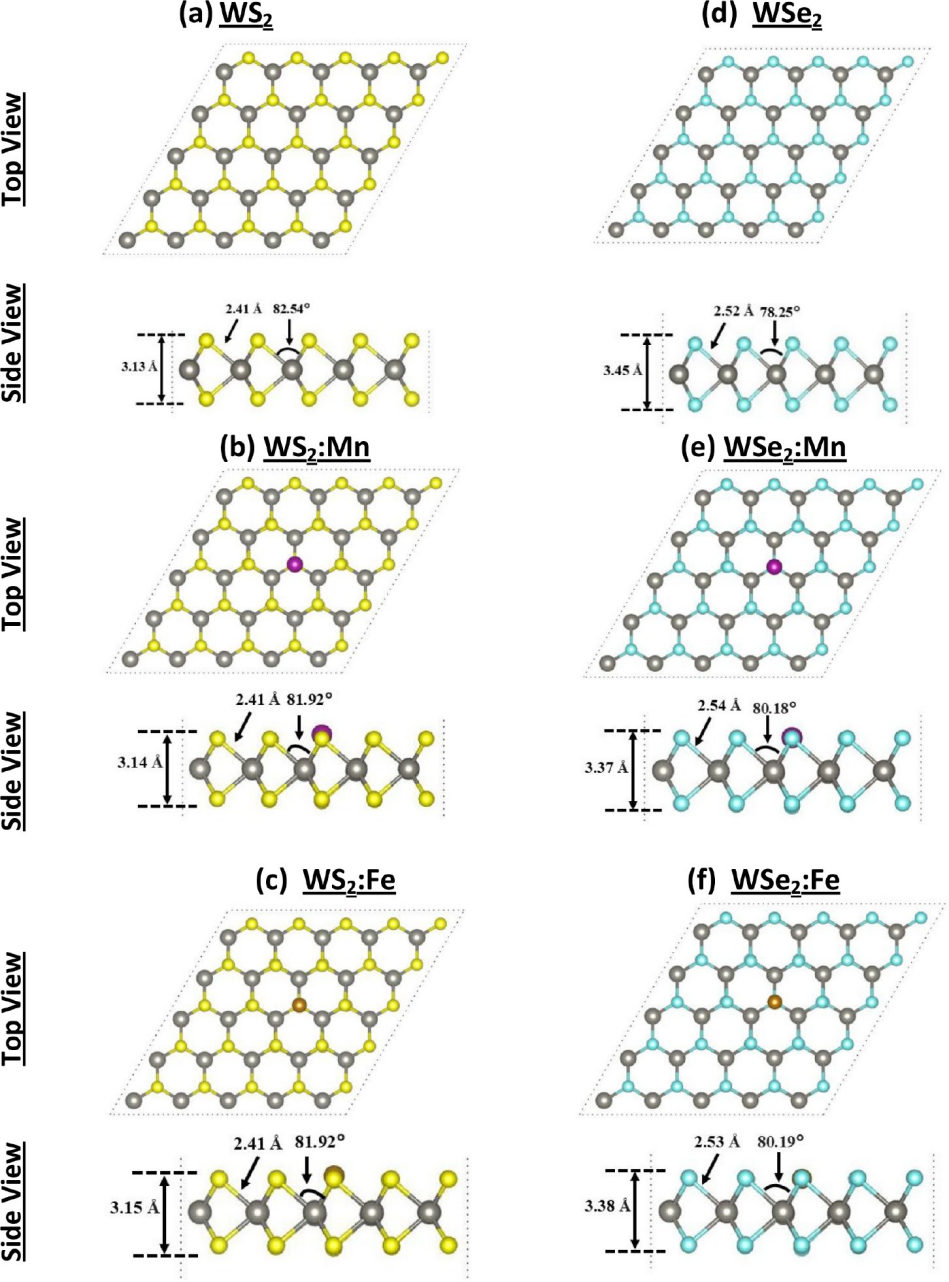
Relaxed atomic structures of pristine and TM-doped TMDs (WX_2_, X = S, Se) with TM = Mn and Fe, (a)­WS_2_, (b) WS_2_:Mn, (c) WS_2_:Fe, (d) WSe_2_, (e) WSe_2_:Mn, and (f) WSe_2_:Fe. Both top and side views are shown. A computational sample of 5 × 5 primitive cells is used.

### TM Doped WX_2_

Based upon our recent investigations, it has been demonstrated that doping with TM atoms should target the anion sites in TMDs (MoX_2_, X = S, Se) to warrant the enhancement in hydrogen storage.^37^ The reason behind this was attributed to the fact that the TM doping of the X-site results in the formation of strong dipole moments and consequently of strong vdW interactions, enhancing the uptake of gravimetric capacity.^37^ Similarly, the idea in our present work is to explore such dipole moments to provide strong physisorption with VOC molecules to the extent of achieving selective adsorption compared to the interfering air molecules. Moreover, to make sure that the TM doping X-site is stable, we have performed ab initio molecular dynamics (AIMD) simulations at 400 K for all the four considered samples (a) WS_2_:Mn, (b) WS_2_:Fe, (c) WSe_2_:Mn, and (d) WSe_2_:Fe. The results are shown in Figure S1. The obtained total energies for these latter samples are about −605.5 eV, −603.5 eV, −559.2 eV, and −557.0 eV, respectively. As shown in Figure S1, the fluctuations of total energy are about 1.0 eV in magnitude or less. Hence, the AIMD simulation has revealed and corroborated the thermodynamic stability of the four doped samples.

Figure 2 and Figure S2 display the spin-polarized band structures and partial and total densities of states (PDOS, TDOS) of pristine and Mn/Fe doped WS_2_ and WSe_2_ monolayers, respectively. The energy range near the Fermi level within 4.0 eV is targeted, as it concerns the physical properties. Pristine WS_2_ and WSe_2_ exhibit paramagnetic semiconducting behavior with direct bandgap energy at the M-point in the Brillouin zone. The calculated bandgap energies are *E*_g_ = 1.81 and 1.54 eV for WS_2_ and WSe_2_ MLs, respectively. These values are in good agreement with the first-principles calculations, for instance, 1.80 and 1.57 eV, reported by Muoi et al.^51^ It is a well-known fact that the use of GGA-PBE underestimates the bandgap, as these latter values are less than the experimental ones, for instance, 2.05 eV^49^ and 1.70 eV.^52^ To improve the theoretical bandgap, one needs to explore further hybrid functionals. Yet, we have already incorporated the GGA-PBE functionals to improve the results, and our current task remains focused on the adsorption properties. Nevertheless, these latter functionals are not needed in our present investigation for two reasons: (i) the theme of our study focuses on the adsorption and gas-sensing properties not the optical ones; (ii) the functionalization of WS_2_ and WSe_2_ with TM atoms, such as TM = Mn and Fe, would metalize the samples. In this regard, the inclusion of the U-Hubbard parameter to take care of the magnetic effects of the dopants becomes more important. As a matter of fact, we have included the default value *U* = −4.5 eV^53^ in all our calculations involving TM atoms.

**Figure 2:**
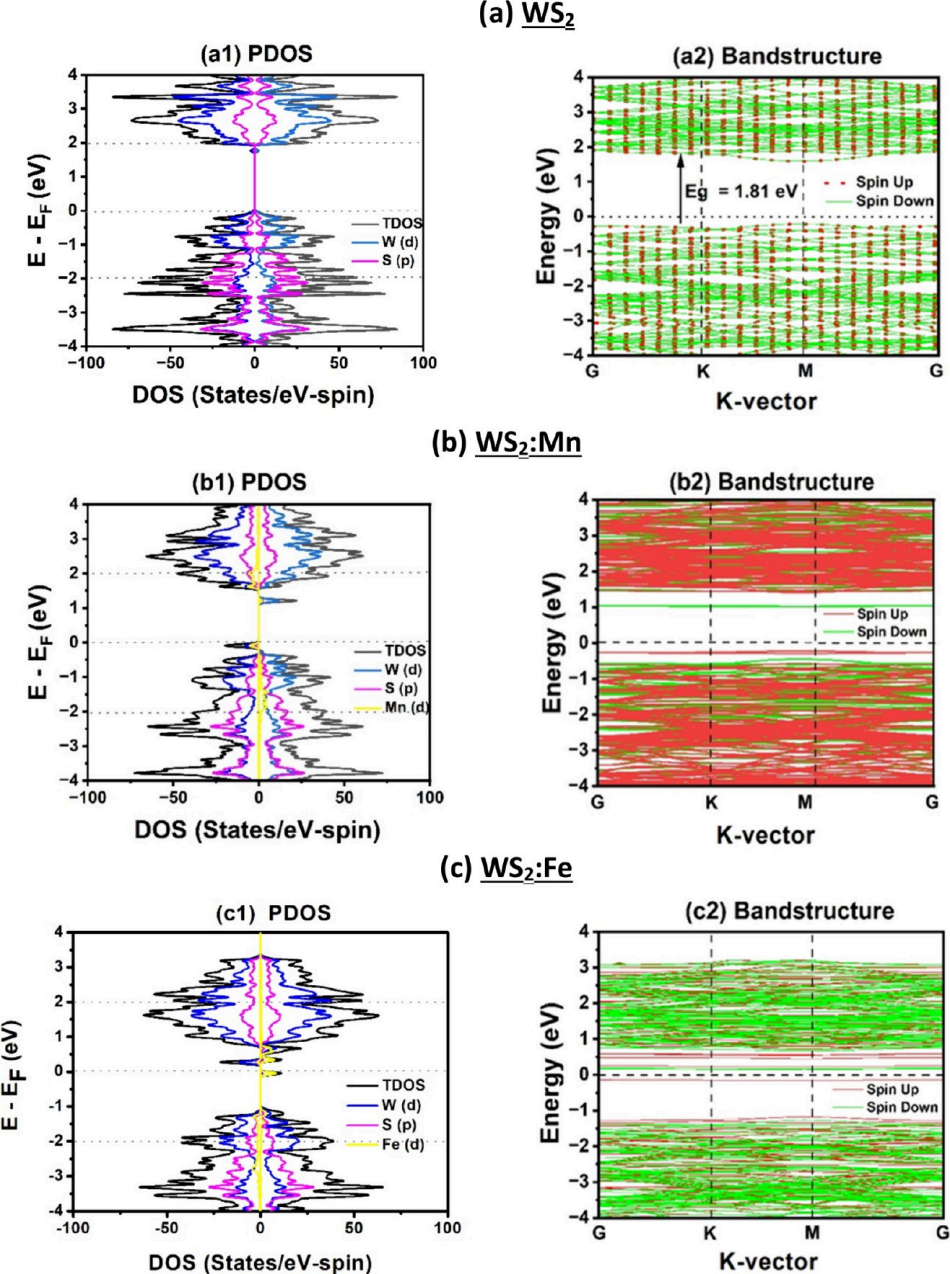
Spin-polarized band structure, PDOS and TDOS for pristine and TM-doped WS_2_: (a) Pristine WS_2_, (b) WS_2_:Mn, and (c) WS_2_:Fe. Fermi level is taken as energy reference (*E*_F_ = 0) and the energy range of [*E*_F_ −4, *E*_F_ +4] eV is shown. In the bands, the spin-up and spin-down states are shown in red and green lines, respectively.

The pristine WX_2_ monolayer exhibits paramagnetic semiconducting behavior where the spin-up and spin-down states are degenerated as shown in Figures 2 and S2a-1,b-1 with symmetric PDOSs. The doping with ferromagnetic transition metal “TM” atoms such as Mn and Fe at the anion site “X” introduces magnetism into the monolayer. Both Mn and Fe alter covalent bonding with the lattice by involving two electrons, but many other d-state electrons remain free in the dangling bonds. As shown in Figures 2 and S2,b-1,2,c-1,2, many peaks and correspondingly flat bands are introduced into the forbidden bandgap and should be attributed to the dangling bonds on TM doping atoms. These dangling bonds will play an essential role in charge exchange with VOCs to produce selectivity, as will be elaborated on in the text below.

### Adsorption of VOCs

To make sure that the large molecules (such as VOCs) achieve global-energy configuration after the process of atomic relaxation, a protocol has been followed in our previous studies on the detection of VOCs related to cancer diseases^34–36^ and hydrogen storage.^37–40^ Some preliminary tests must be carried out as follows. A small-sized molecule such as N_2_ was relaxed on the TM-dopant site starting from a height of about 2.0 Å. Under the field of van der Waals interactions, usually, the process of relaxation should follow the Lennard-Jones profile with an optimal distance that can be more or less than 2.0 Å. In the next step of the challenge, in dealing with large molecules, tests must be conducted on the effect of the initial configuration by paying attention to the two factors concerning the initial adsorption distance and the initial molecule orientation. Usually, the global minimum-energy configuration can be achieved, and it corresponds to the one on top of the dopant and with the molecular orientation being parallel to the adsorbent where the molecule optimizes its interactions.

Four adsorbent samples have been considered WX_2_:TM@X (with X = S, Se and TM = Mn, Fe) to study the adsorption of five VOCs associated with LC. Figure 3a,b shows the results of relaxed atomic structures of the five VOCs on the two samples (a) WS_2_:Mn and (b) WS_2_:Fe, whereas Figure S3 shows the results on the other two samples (a) WSe_2_:Mn and (b) WSe_2_:Fe. Both the top and side views are displayed for the sake of clarity. Figure 3 and Figure S3 show that all the adsorptions of the five VOCs are at the border between strong physisorption and weak chemisorption with no evidence of occurrence of dissociation. Yet, the strength of the interaction should be proportional to the adsorption distance, energy, and amount of charge exchanged.

**Figure 3:**
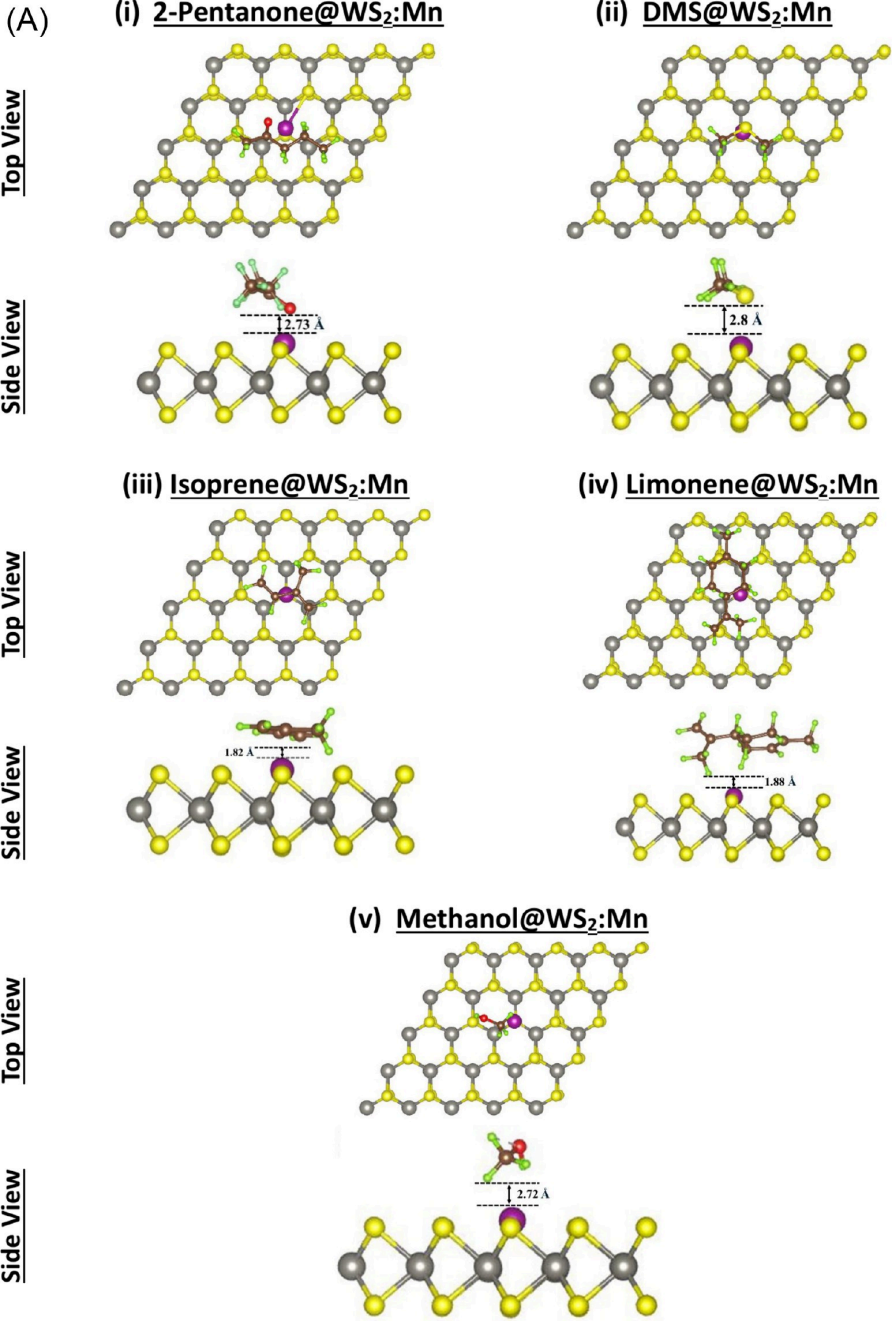

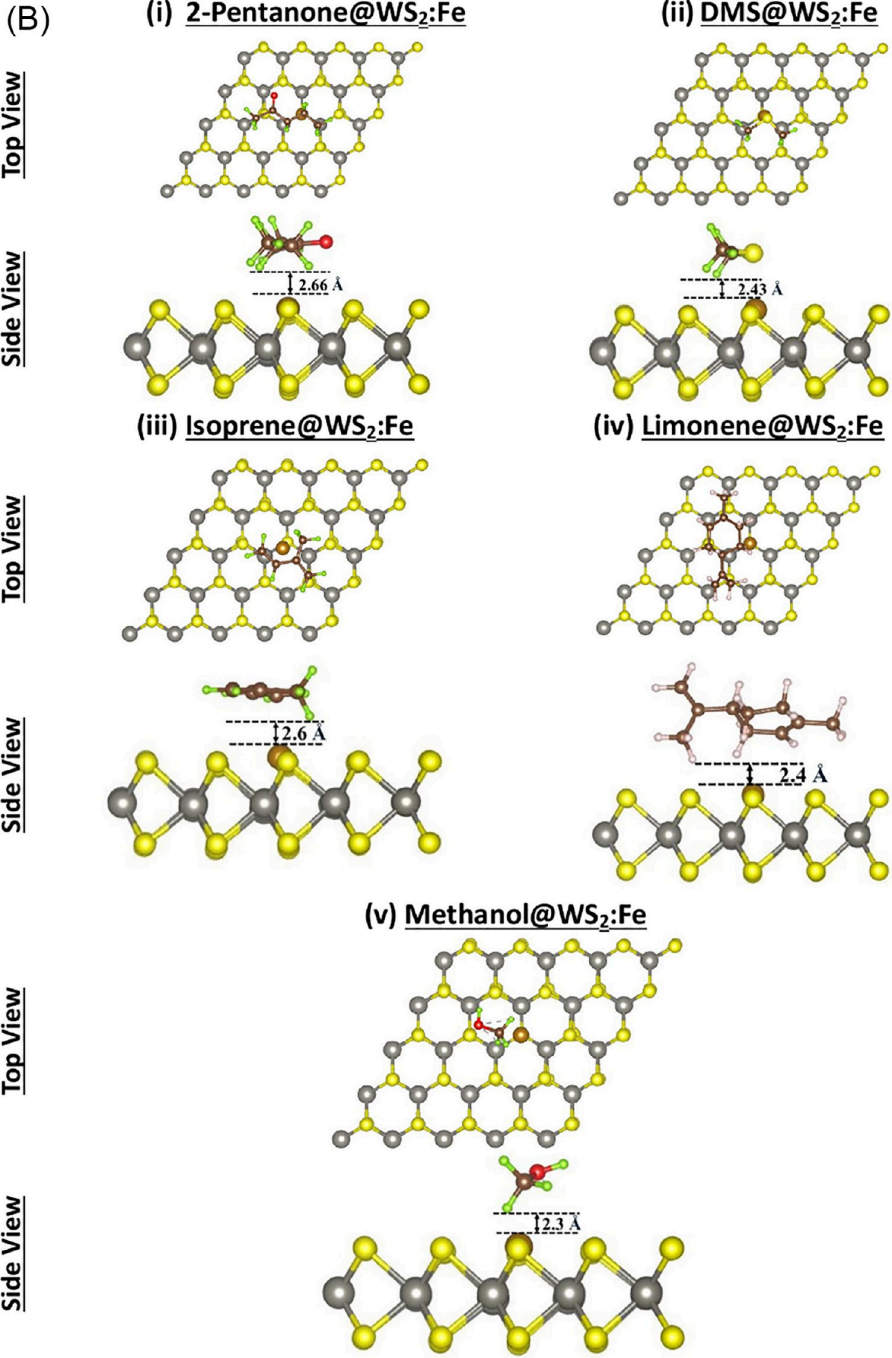
Relaxed atomic structures after the adsorption of five VOCs related to liver cirrhosis biomarkers on two samples: (A) WS_2_:Mn and (B) WS_2_:Fe. Atomic species are colored as follows: W (gray), S (yellow), Mn (purple), Fe (big brown), C (small brown), H (green), and O (red).

Figure 3A shows the adsorption distances between Mn and five VOCs to be 2.73, 2.80, 1.82, 1.88, and 2.72 Å, respectively. So, the strong adsorption seems to take place between VOC-3 (Isoprene) as well as VOC-4 (Limonene) and the adsorbent (WS_2_:Mn@S). Figure 3B shows the adsorption distance between Fe and the respective five VOCs to be 2.66, 2.43, 2.60, 2.40, and 2.30 Å. So, all the interactions seem to be physisorption-like and of pure vdW type. Figure S3A displays the adsorption distance between Mn doping WSe_2_ and the five VOCs to be 2.68, 2.40, 1.85, 1.96, and 2.20 Å, respectively. So, again VOC-3 (Isoprene) and VOC-4 (Limonene) exhibit relatively strong adsorptions on the adsorbent WSe_2_:Mn@Se. On the last sample WSe_2_:Fe@Se, Figure S3B shows the adsorption distances of the five VOCs to be 2.30, 2.10, 1.80, 2.40, and 1.80 Å, respectively. The results reveal that a bit stronger adsorption took place on WSe_2_:Fe compared to that on WS_2_:Fe. Meanwhile, in the former sample, VOC-3 (Isoprene) and VOC-5 (Methanol) seem to alter stronger interactions with the adsorbent compared to the other three VOCs.

Figure 4 shows the absolute values of adsorption energy |*E*_ads_|, charge transfer |Δ*q*|, and change of magnetization |Δ*M*| due to the adsorption of the five VOCs and four interfering air molecules on the four samples: (a)­WS_2_:Mn, (b) WS_2_:Fe, (c) WSe_2_:Mn, and (d) WSe_2_:Fe. One can notice the following trends, which are in support of adsorbent being selective toward the adsorptions of five VOCs. (i) All five VOCs have strong adsorption energies much higher than 1.00 eV, which is recommended for selectivity, while the |*E*_ads_| of interfering air molecules do not exceed 0.5 eV/molecules. (ii) The charge transferred from the TM catalyst to VOCs is also proportional to *|E*_ads_| and ranges from 0.4 to 0.8 e, while |Δ*q*| does not exceed 0.15 e in the case of interfering air molecules. (iii) The magnetization is associated with the adsorption of VOCs but does not exist even in the case of adsorption of interfering air molecules.

**Figure 4:**
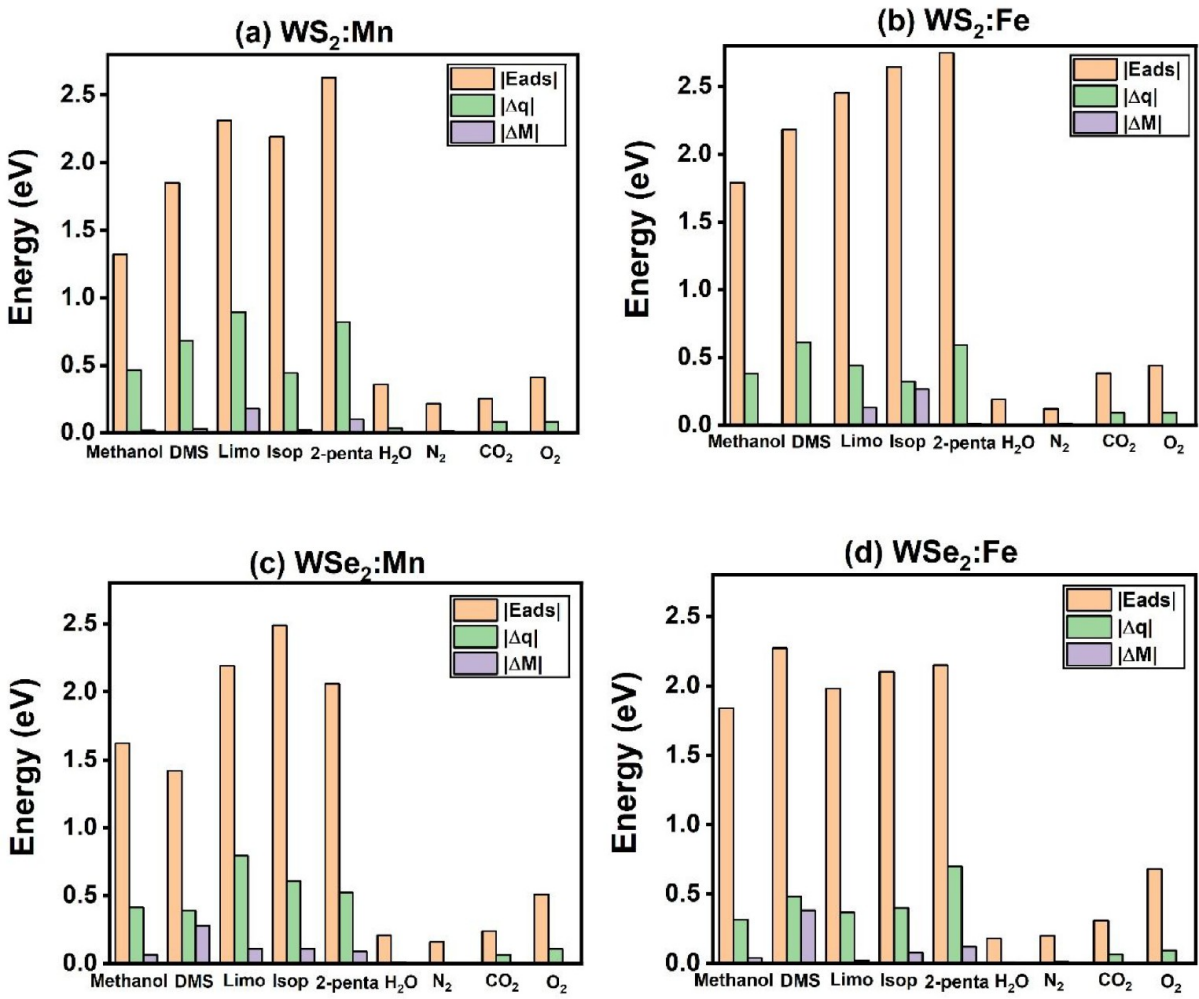
Absolute values of adsorption energy, charge transfer, and change in magnetization due to the adsorption of nine molecules (5 VOCs and 4 air molecules) on (a) WS_2_:Mn, (b) WS_2_:Fe, (c) WSe_2_:Mn, and (d) WSe_2_:Fe.

To further analyze the selective adsorption of the four TM-doped samples (WX_2_:TM, X = S, Se and TM = Mn, Fe) toward the VOCs, the nature of interaction should come into interplay for inspection. We define the ratio of contribution of the vdW interactions into the total energy^54^ as follows:

$$R=\frac{\left|E_{tot}^{vdW}-E_{tot}^{NovdW}\right|}{\left|E_{tot}^{vdW}\right|}\times 100\%$$ 3

where *E*_tot_^vdW^ and *E*_tot_^NovdW^ stand for the total energies with inclusion/exclusion of vdW interactions, respectively. The results, displayed in Figure 5, show that all of the adsorption energies of the five studied VOCs on the four doped samples have a contribution from vdW interactions exceeding 60%. The values of adsorption energy, charge exchange, and change in magnetization are also displayed in Table S1. The strong vdW character of the interactions with VOCs displayed in Figure 5 reveals that the interactions do remain within the limits of strong physisorption, which can cause a charge transfer in the range of |Δ*q*| = 0.4–0.8 e, while the interactions with the air molecules cause a much smaller charge transfer of |Δ*q*| < 0.1 e.

**Figure 5:**
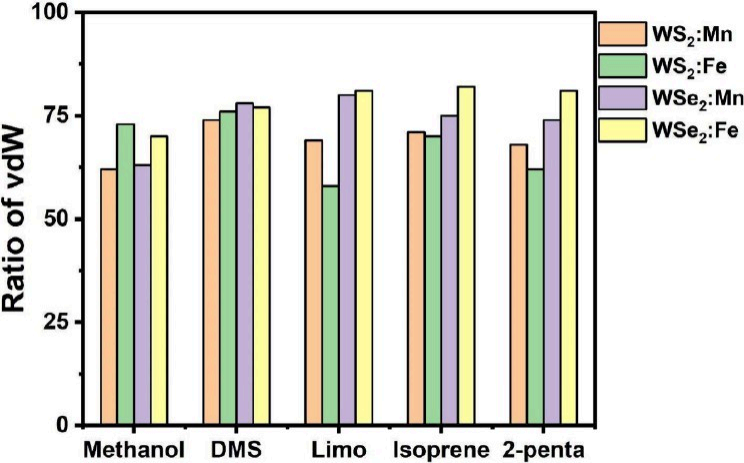
Ratio of contribution of vdW interaction energy to the total adsorption energy of five VOCs on four samples (WX_2_:TM, X = S, Se and TM = Mn, Fe).

### Electronic Properties

Figure 6 shows the electronic properties through the spin-polarized band structures and PDOS/TDOS for the two samples studied in Figure 4 after the adsorption of the five VOCs, while Figure S4 displays the electronic structures of samples studied in Figure S3. The bands corresponding to the spin-up and spin-down states are colored red and green, respectively. The partial DOS of the adsorbent and adsorbate are shown in blue and pink colors, respectively, whereas the TDOS is in the black curve. Figure 5 shows the results corresponding to the adsorptions of five VOCs on the four samples: (WX_2_:TM, X = S, Se and TM = Mn, Fe). One can notice the following trends: (i) Some localized gap states below the Fermi level associated with the substrate were introduced after the adsorption with VOCs. These states play the role of donor states to give charges to the VOC molecules. (ii) The effect of VOC interaction on the DOS is drastic in some cases such as VOC-3 (Isoprene) and VOC-4 (Limonene) on the WS_2_:Fe sample (see Figure 6Bc,d). (iii) The VOCs’ interactions with the adsorbent on the DOS can affect the states near the Fermi level, including the extended states that alter the conductivity, and this will in turn affect the sensor response. In many cases, the many gap states get close to the conduction-band minimum “CBM”, together with bandgap reduction, for instance, (1) VOC-3 (Isoprene) on WS_2_:Mn (Figure 6Ac), (2) VOC-1 (2-pentanone), VOC-2 (DMS), and VOC-4 (Limonene) on WS_2_:Fe (Figure 5), and (3) all five VOCs on WSe_2_:Fe.

**Figure 6:**
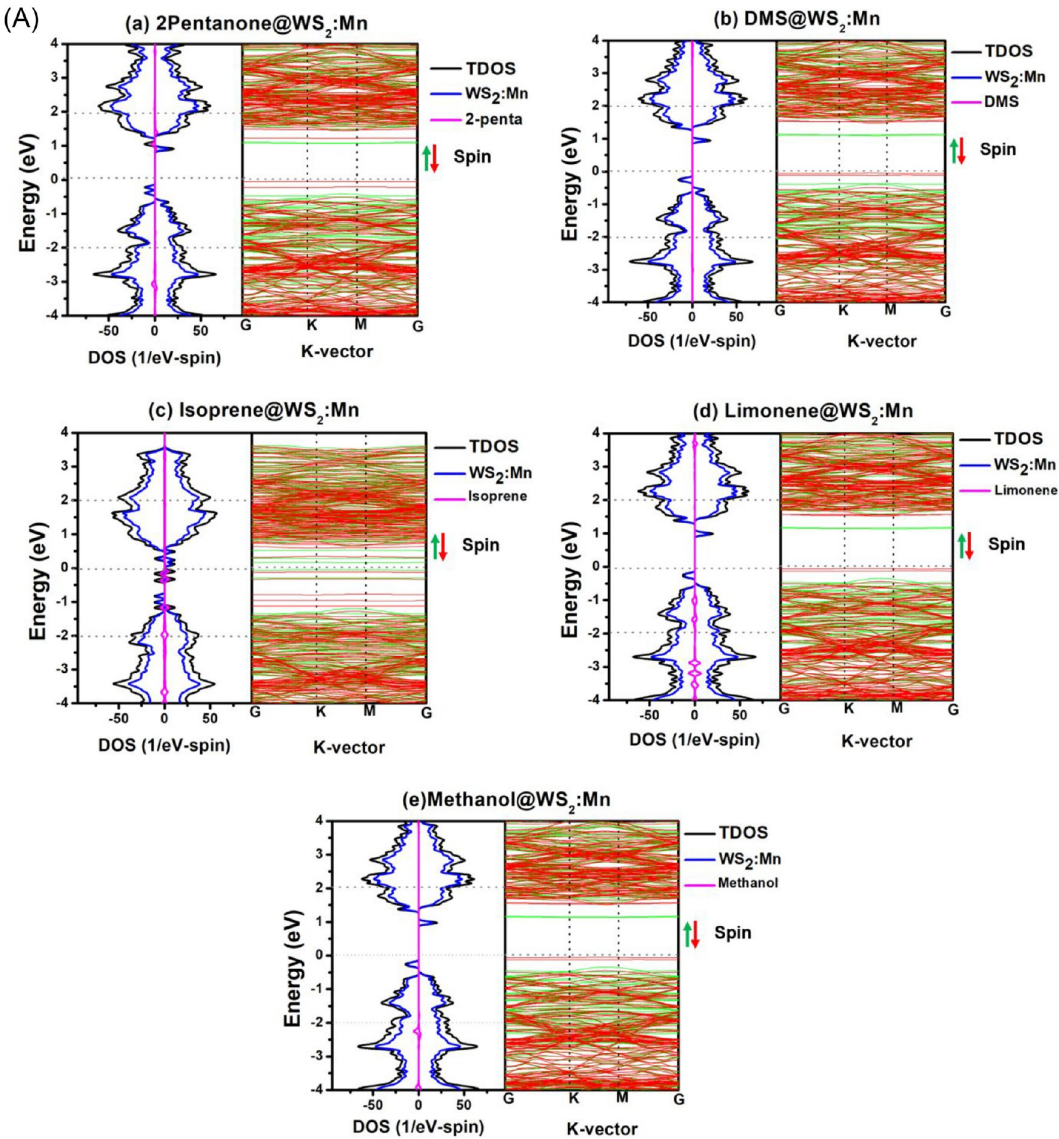

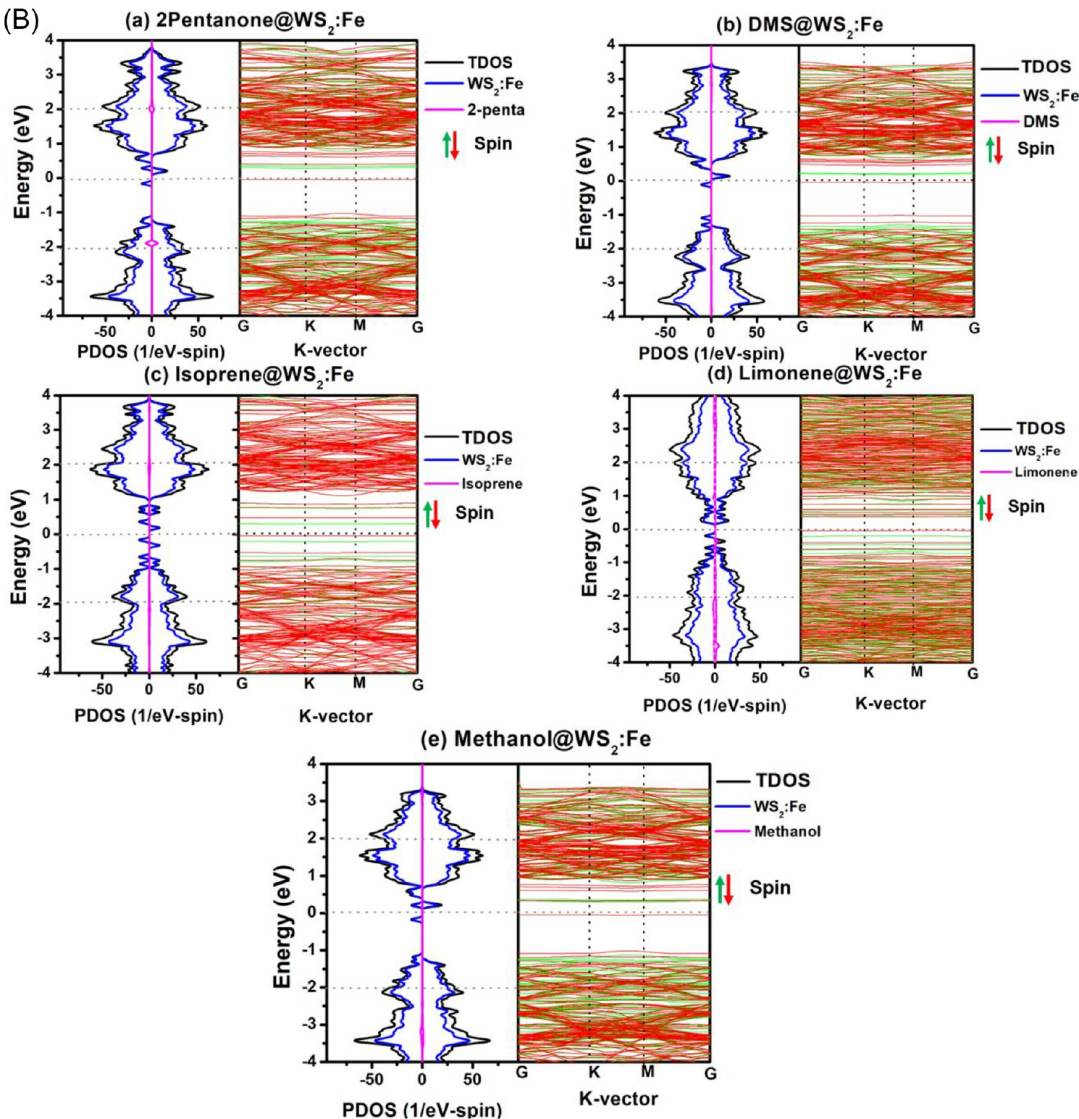
Band structures, PDOS, and TDOS of five VOCs adsorbed on two samples. (A) WS_2_:Mn: (a) 2-Pentanone@WS_2_:Mn, (b) DMS@WS_2_:Mn, (c) Isoprene@WS_2_:Mn, (d) Limonene@WS_2_:Mn, and (e) Methanol@WS_2_:Mn; (B) WS_2_:Fe: (a) 2-Pentanone@WS_2_:Fe, (b) DMS@WS_2_:Fe, (c) Isoprene@WS_2_:Fe, (d) Limonene@WS_2_:Fe, and (e) Methanol@WS_2_:Fe.

### Charge-Density Difference (CDD)

There are two methods to evaluate the charge transfer between adsorbate and adsorbent. Both methods are incorporated into VASP. The first method relies on the Bader charge analysis with the results displayed in Figure 4 and Table S1. The second method relies on the charge-density difference (CDD) plots shown in Figure 7 and Figure S5 for samples related to WS_2_ and WSe_2_, respectively. The charge gain (deficit) is shown in a yellow (canyon) color. The results are shown after the adsorption of the five VOCs on the four samples: (a) WS_2_:Mn, (b) WS_2_:Fe, (c) WSe_2_:Mn, and (d) WSe_2_:Fe. The common trend in Figure 7 and Figure S5 is the accumulation of charge along the bond between VOC and TM, representing under certain strong adsorption conditions the formation of a covalent bond with partial ionic characters. Many parts of the VOC molecules look depleted from the charge, as they correspond to hydrogen bonds. Of course, charge always gets accumulated at atomic sites with high electronegativity characters (χ° = 3.44 > χ^S^ = 2.58 > χ^C^ = 2.55 > χ^H^ = 2.2 > χ^Fe^ = 1.83 > χ^Mn^ = 1.55 Pauling units).^55^

**Figure 7:**
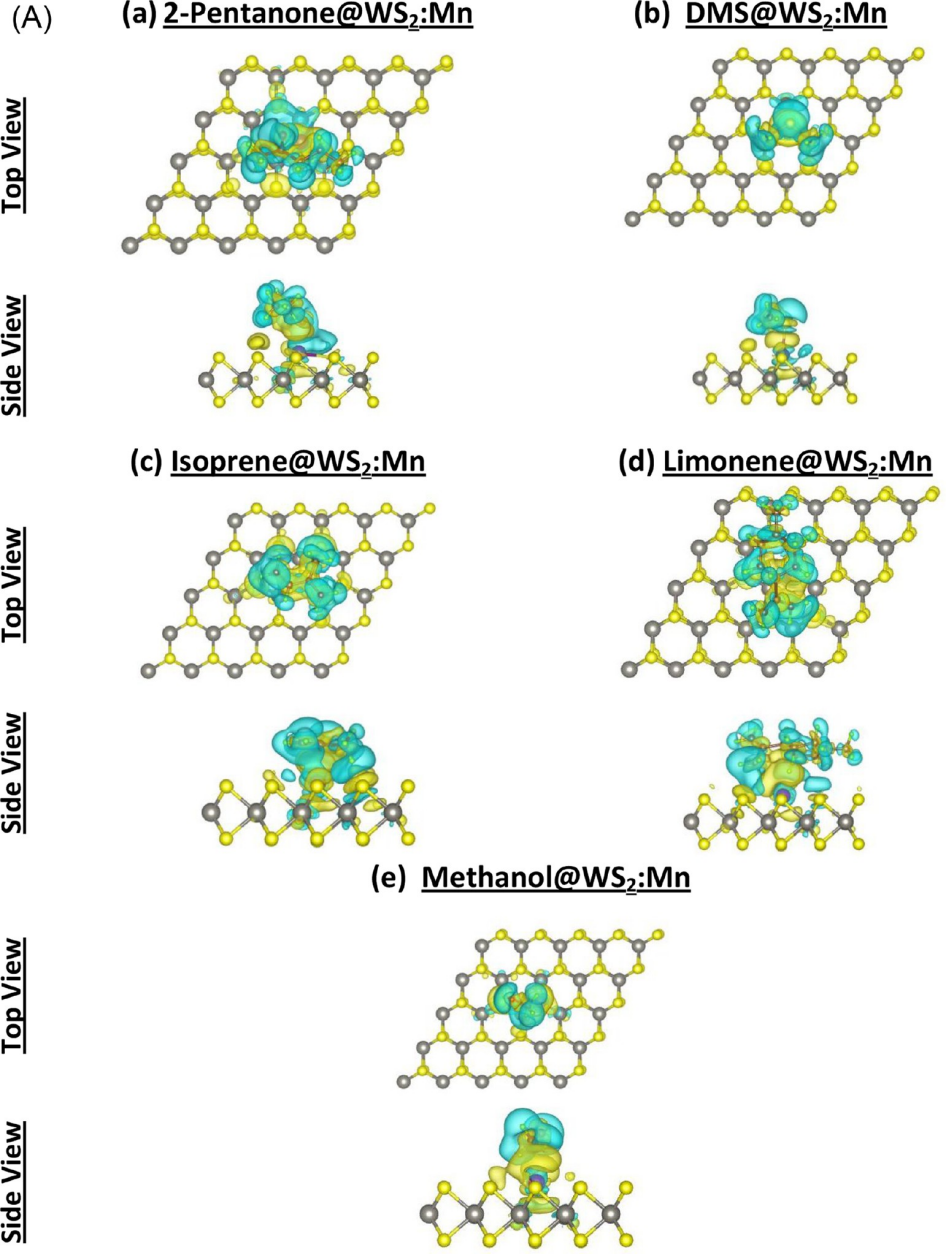

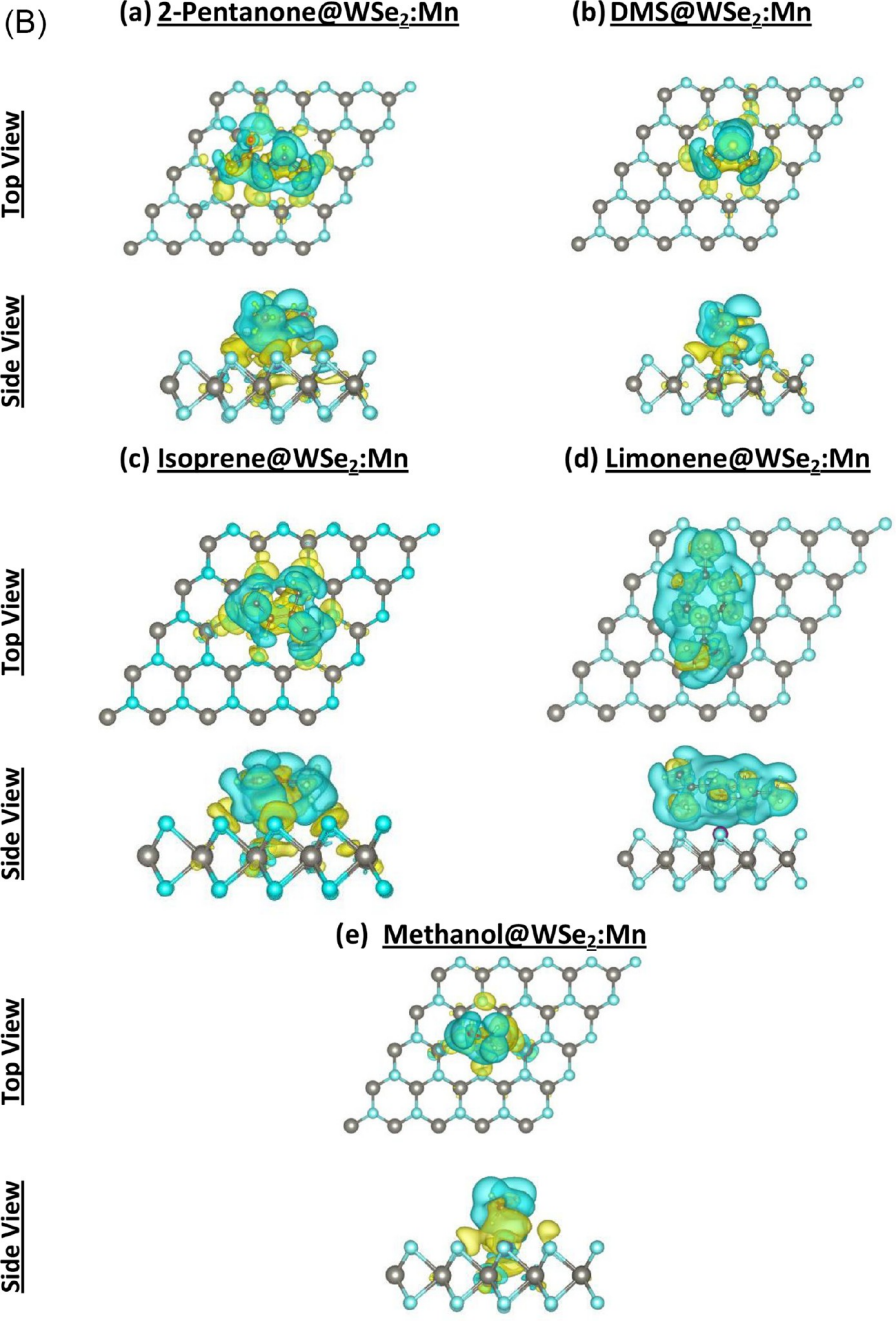
Charge density difference (CDD) due to the adsorptions of five VOC molecules on two samples. (A) WS_2_:Mn: (a) 2-Pentanone@WS_2_:Mn, (b) DMS@WS_2_:Mn, (c) Isoprene@WS_2_:Mn, (d) Limonene@WS_2_:Mn, and (e) Methanol@WS_2_:Mn; (B) WS_2_:Fe: (a) 2-Pentanone@WSe_2_:Mn, (b) DMS@WSe_2_:Mn, (c) Isoprene@WSe_2_:Mn, (d) Limonene@WSe_2_:Mn, and (e) Methanol@WSe_2_:Mn.

### Work Function and Sensor Response

Figure 8 shows the results of the work function Φ calculated for nine molecules (five VOCs and four air molecules) before and after the adsorption processes on the four samples: (a) WS_2_:Mn, (b) WS_2_:Fe, (c) WSe_2_:Mn, and (d) WSe_2_:Fe. While all four samples possess metallic behaviors, the change in work function ΔΦ should reveal a drastic change in the electronic structure at the Fermi level and consequently a great effect on the conductivity and the sensor response. Figure 8 clearly shows that ΔΦ corresponding to the interfering air molecules (N_2_, O_2_, H_2_O and CO_2_) is much smaller than those corresponding to the five VOCs cases.

**Figure 8:**
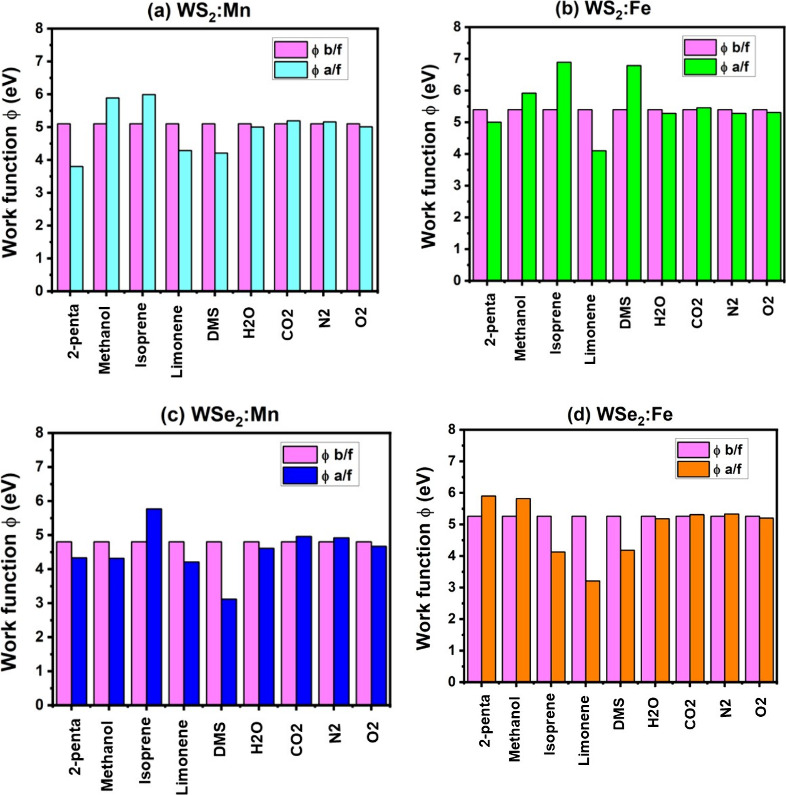
Work function before and after the adsorption of nine molecules (5 VOCs and 4 air molecules) on 4 samples: (a) WS_2_:Mn, (b) WS_2_:Fe, (c) WSe_2_:Mn, and (d) WSe_2_:Fe.

From the perspective of work function, the sensor response has been defined^56^ as follows:

$$S=\frac{\left|\Phi_{f}-\Phi_{i}\right|}{\left|\Phi_{i}\right|}\times 100\%$$ 4

where Φ_i_ and Φ_f_ are the work functions before and after the molecular adsorptions, respectively. Figure 9 shows the results of sensitivity (i.e., sensor response) for all nine molecules on the four samples. Figure 9 clearly shows the selectivity of all four samples towards the detection of the five VOCs.

**Figure 9:**
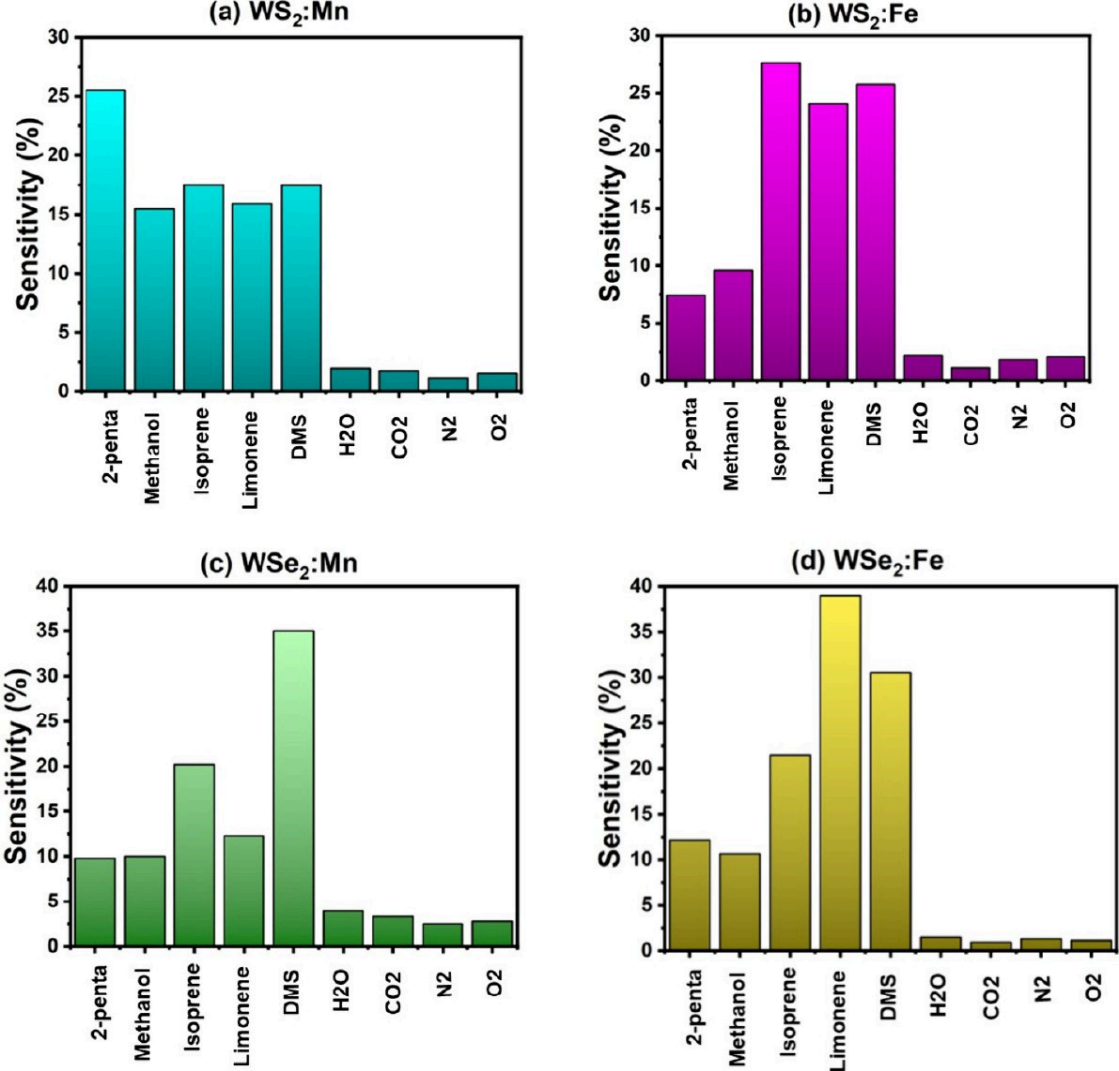
Sensor responses of the adsorption of nine molecules (5 VOCs and 4 air molecules) on four samples: (a) WS_2_:Mn, (b) WS_2_:Fe, (c) WSe_2_:Mn, and (d) WSe_2_:Fe.

## Conclusions

The state-of-the-art computational method based on spin-polarized DFT with the inclusion of vdW interaction was explored to study the adsorption properties of VOCs related to liver cirrhosis (LC) in comparison with those of interfering air molecules. This study aims to investigate the adsorption mechanism of TM-functionalized WS_2_ and WSe_2_ monolayers toward the detection of LC-related VOCs, namely, 2-pentanone, dimethyl sulfide, isoprene, limonene, and methanol. We found that the Mn and Fe doping of the chalcogen sites in WX_2_ significantly enhanced the adsorption energy of VOCs compared to that of interfering air molecules, leading to clear selectivity. Additionally, magnetism was introduced into the monolayer through Mn and Fe doping, and notable changes in magnetization were observed only during interactions with VOCs, an effect expected to influence the IV characteristics and, consequently, the sensor response. The density of states (DOS) analysis revealed pronounced changes near the Fermi level upon VOC adsorption, further impacting conductivity and sensor performance. A complementary pathway to achieving selectivity was demonstrated through variations in work function, which showed substantially larger shifts for VOCs than for air molecules. Our results propose WX_2_:TM (X = S, Se with TM = Mn, Fe) to be considered as a strong candidate material for a platform of biosensors with high selectivity toward the detection of LC biomarkers. This work uncovers atomistic details of adsorption behavior, charge redistribution, and changes in electronic structure, helping to inform and inspire future experimental progress in the design of nanobiosensors.

## Supplementary Material



## Notes

AIMD simulations @ 400 K showing thermodynamic stability of WS_2_:Mn, WS_2_:Fe, WSe_2_:Mn, and WSe_2_:Fe samples; Spin-polarized band structure and TDOS of WS_2_:Mn, WS_2_:Fe, WSe_2_:Mn, and WSe_2_:Fe samples; Relaxed configurations of 5 VOCs related to liver cirrhosis biomarkers on WSe_2_:Mn and WSe_2_:Fe samples; Spin-polarized band structure and TDOS of 5 VOCs related to liver cirrhosis biomarkers on WSe_2_:Mn and WSe_2_:Fe samples; Charge-density difference “CDD” of all the previous 10 samples corresponding to 5 VOCs adsorbed on WSe_2_:Mn and WSe_2_:Fe samples; Table showing adsorption data (*E*_ads_, Δ*q*, *M*, τ) of 5 VOCs and 4 interfering air molecules on WS_2_:Mn, WS_2_:Fe, WSe_2_:Mn, and WSe_2_:Fe samples (PDF)

## Abbreviations

DFT: density functional theory
VASP: Vienna ab initio simulation package
AIMD: ab initio molecular dynamics
GGA: general gradient approximation
PBE: Perdew–Burke–Ernzerhof hybrid functional
HSE06: Heyd–Scuseria–Ernzerhof hybrid functional with a 06 parametrization
LC: liver cirrhosis
VOCs: volatile organic compounds
TMDs: transition metal dichalcogenides
TM: transition metal atom
vdW: van der Waals interactions
WS_2_: tungsten disulfide
WSe_2_: tungsten diselenide
NASH: nonalcoholic steatohepatitis
e-Nose: electronic nose
*E*_g_: bandgap energy
*E*_F_: Fermi energy
*E*_ads_: adsorption energy
*E*_bind_: binding energy
*E*_coh_: cohesive energy
DOS: density of states
TDOS: total density of states
PDOS: projected or partial density of states
ppm: part per million
ppb: part per billion
S: sensor response or sensitivity
PC: primitive cell
SI: Supporting Information
