# Supplementary material for: Sensitive Detection of Specific Volatile Organic Compounds by Functionalized Transition Metal Dichalcogenide Monolayers
Source: Langmuir. 2025 Aug 12;41(33):22455–70. doi: 10.1021/acs.langmuir.5c02852 (PMC12392728; doi:10.1021/acs.langmuir.5c02852)
Supplement: Supplementary file 1 [file la5c02852_si_001.pdf]

Supporting Information

Sensitive Detection of Specific Volatile Organic Compounds by Functionalized Transition Metal Dichalcogenides Monolayers

Saba Khan[a], Tanveer Hussain[b], Chandra Veer Singh[a,$], Nacir Tit[c,d,$]

[a] Department of Materials Science and Engineering, University of Toronto, Toronto, Ontario M5S 3E4, Canada

[b] School of Science and Technology, University of New England, Armidale, New South Wales 2351, Australia

[c] Department of Physics, College of Science, UAE University, P.O. Box 15551, Al-Ain, United Arab Emirates

[d] National Water and Energy Centre, UAE University, P.O. Box 15551, Al-Ain, United Arab Emirates

($) Corresponding Authors: chandraveer.singh@utoronto.ca (C.V. Singh), ntit@uaeu.ac.ae (N. Tit)

SUPPLEMENTARY INFORMATION

S1 AIMD simulations held at 400 K on WS_2:Mn, WS_2:Fe, WSe_2:Mn, and WSe_2:Fe.

S2 Spin-polarized band structure, PDOS and TDOS for pristine and TM(Mn/Fe)-doped WSe_2.

S3 Relaxed atomic structures after the adsorption of five VOCs related to liver cirrhosis biomarkers on (A) WSe_2:Mn, and (B) WSe_2:Fe.

S4 Band structures and PDOS/TDOS of five VOCs adsorbed on two samples: (A) WSe_2:Mn and (B) WSe_2:Fe.

S5 Charge density difference (CDD) due to the adsorptions of five VOC molecules on two samples: (A) WSe_2:Mn and (B) WSe_2:Fe.

T1 Results of adsorption energy, charge transfer and change of magnetization due to the adsorption of 9 molecules (5 VOCs and 4 air molecules) on (a) WS_2:Mn, (b) WS_2:Fe, (c) WSe_2:Mn, and (d) WSe_2:Fe.

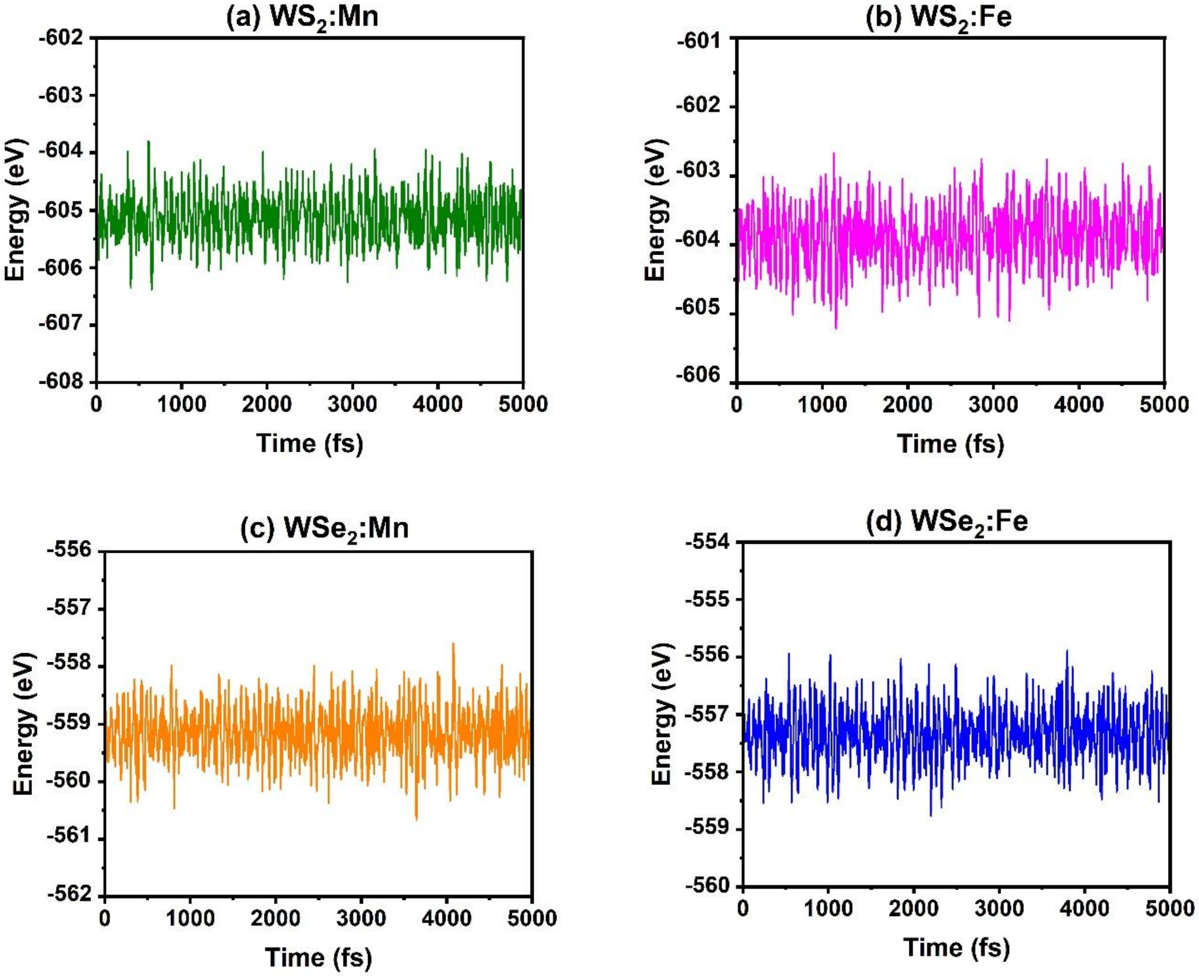

Figure S1: AIMD simulations held at 400 K on the four samples: (a) WS_2:Mn, (b) WS_2:Fe, (c) WSe_2:Mn, and (d) WSe_2:Fe. The simulations corroborate the thermodynamic stability of the four samples.

(a) <u>WSe_2</u>

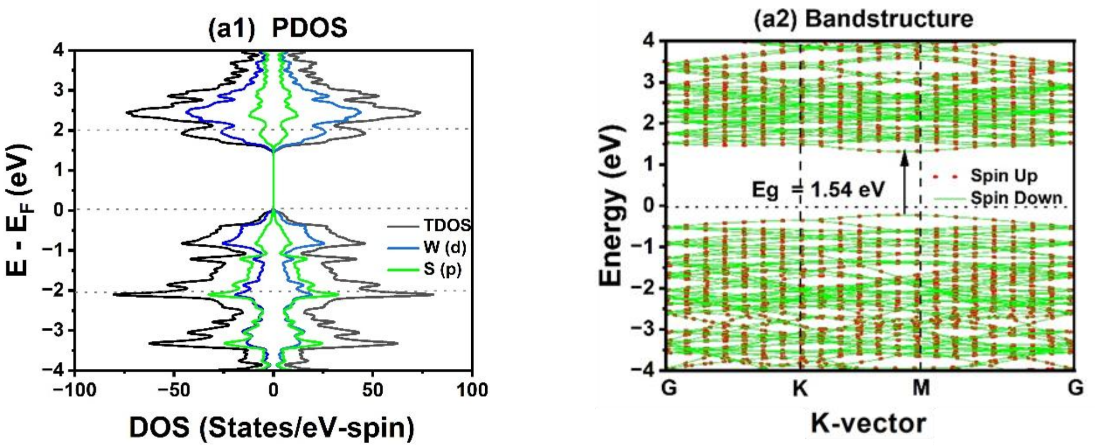

(b) <u>WSe_2:Mn</u>

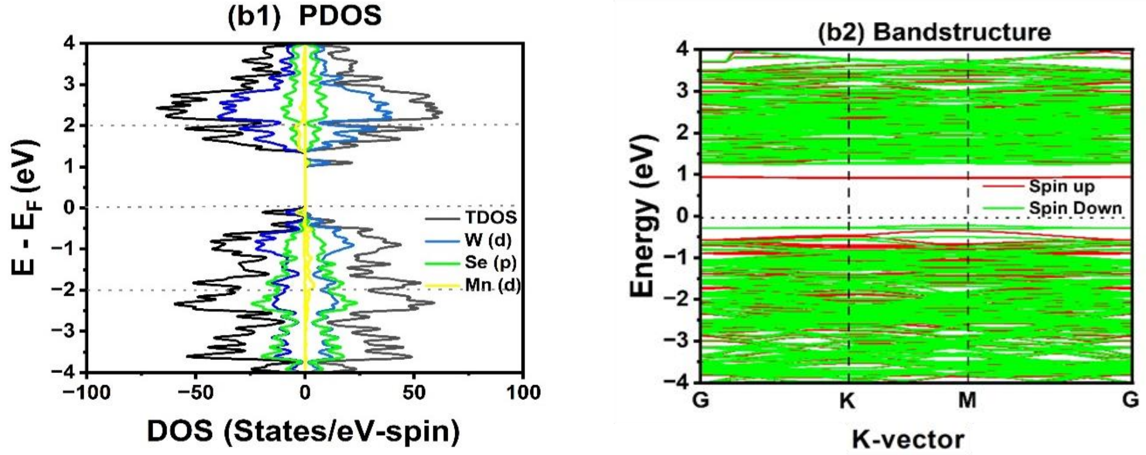

(c) <u>WSe_2:Fe</u>

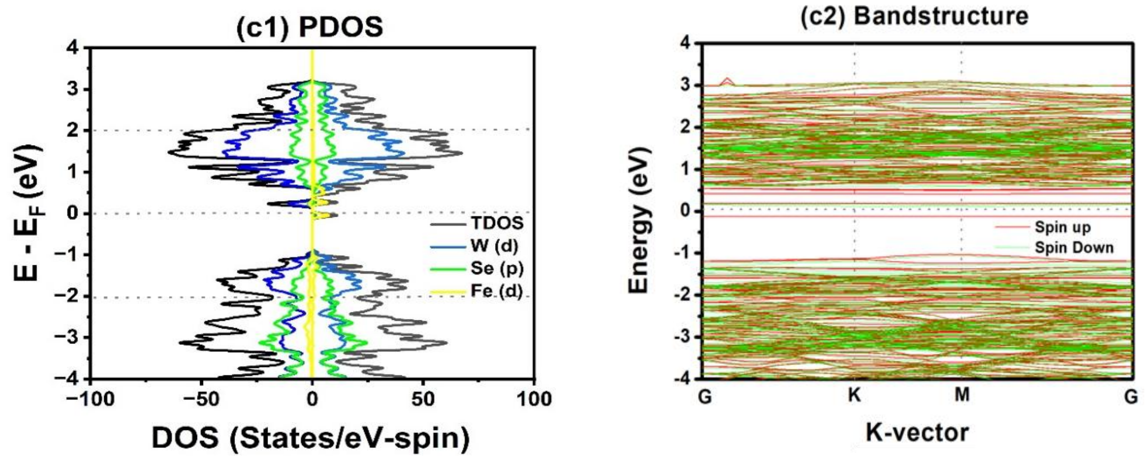

Figure S2: Spin-polarized band structure, PDOS and TDOS for (a) pristine and TM-doped WSe_2: (b) TM = Mn and (c) TM = Fe. Fermi level is taken as energy reference ($E_F = 0$) and energy range [E_F-4, E_F+4] eV is shown. In the bands, the spin-up and spin-down states are shown in red and green colors, respectively.

(A)

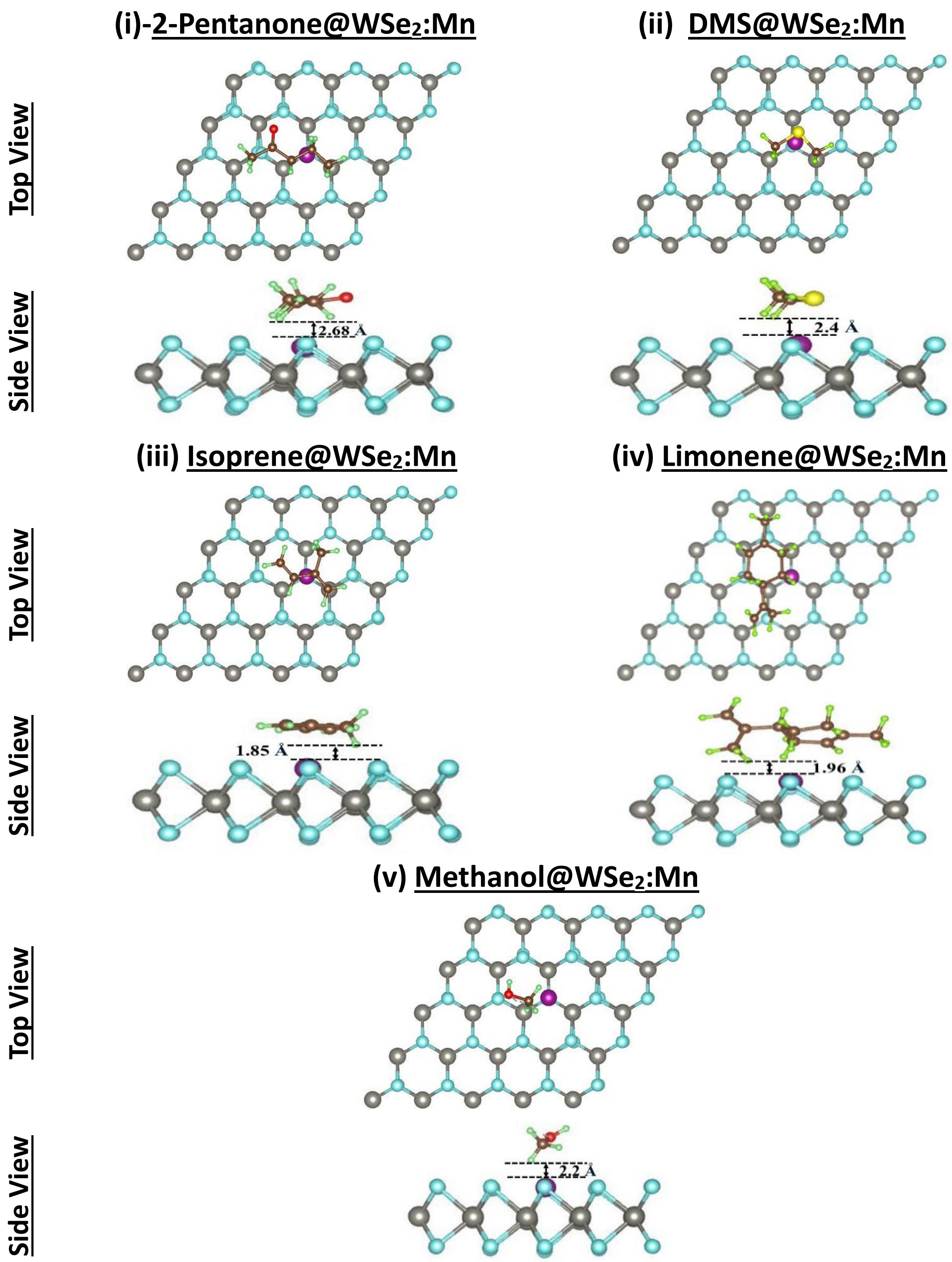
(i)-2-Pentanone@WSe₂:Mn
(ii) DMS@WSe₂:Mn
Top View
Side View
2.68 Å
2.4 Å
(iii) Isoprene@WSe₂:Mn
(iv) Limonene@WSe₂:Mn
1.85 Å
1.96 Å
(v) Methanol@WSe₂:Mn
2.2 Å

(B)

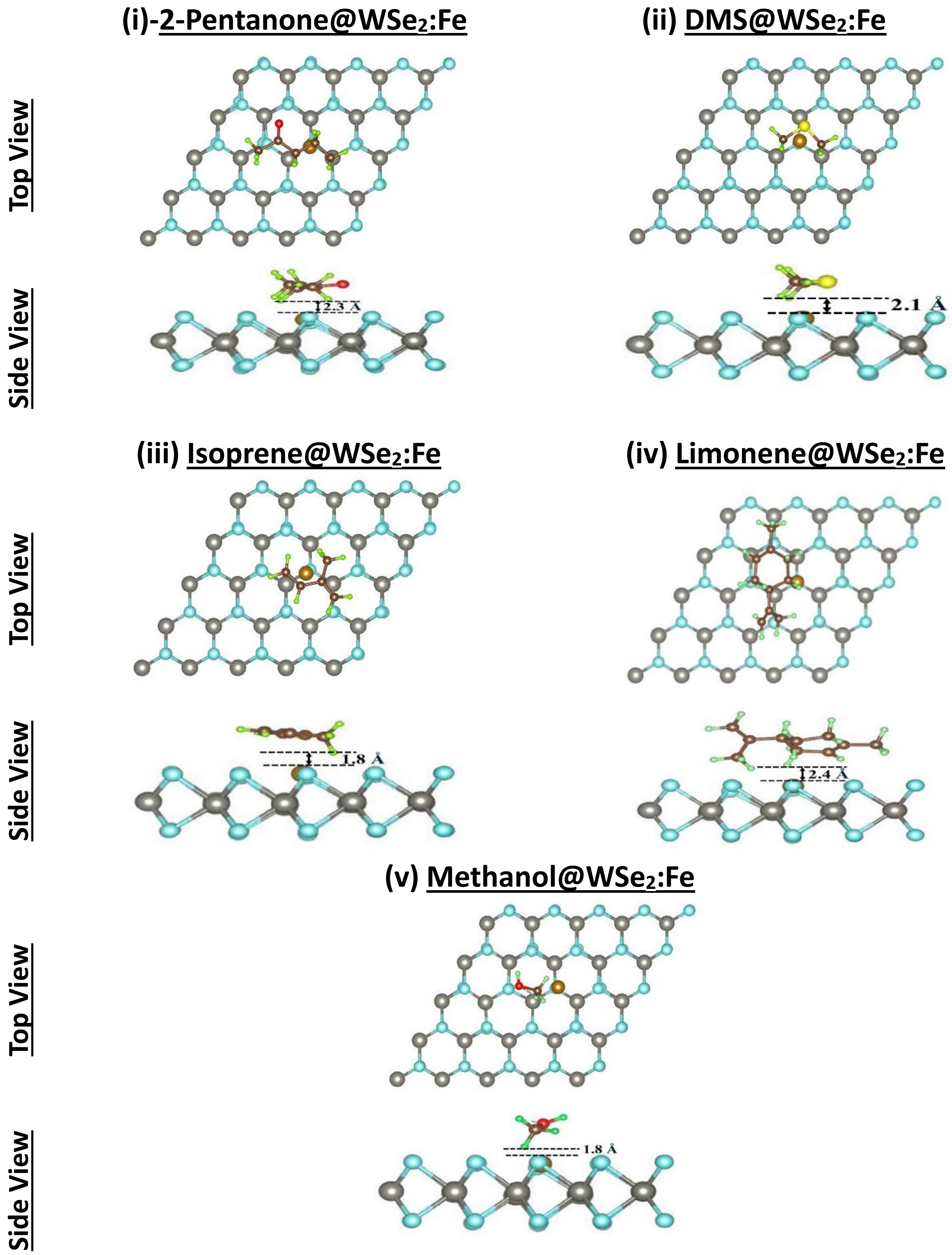

Figure S3: Relaxed atomic structures after the adsorption of five VOCs related to liver cirrhosis biomarkers on two samples: (A) WSe_2:Mn, and (B) WSe_2:Fe. Atomic colors are: W (grey), S (yellow), Se (blue), Mn (purple), Fe (big brown), C (small brown), H (green), O (red).

(A)

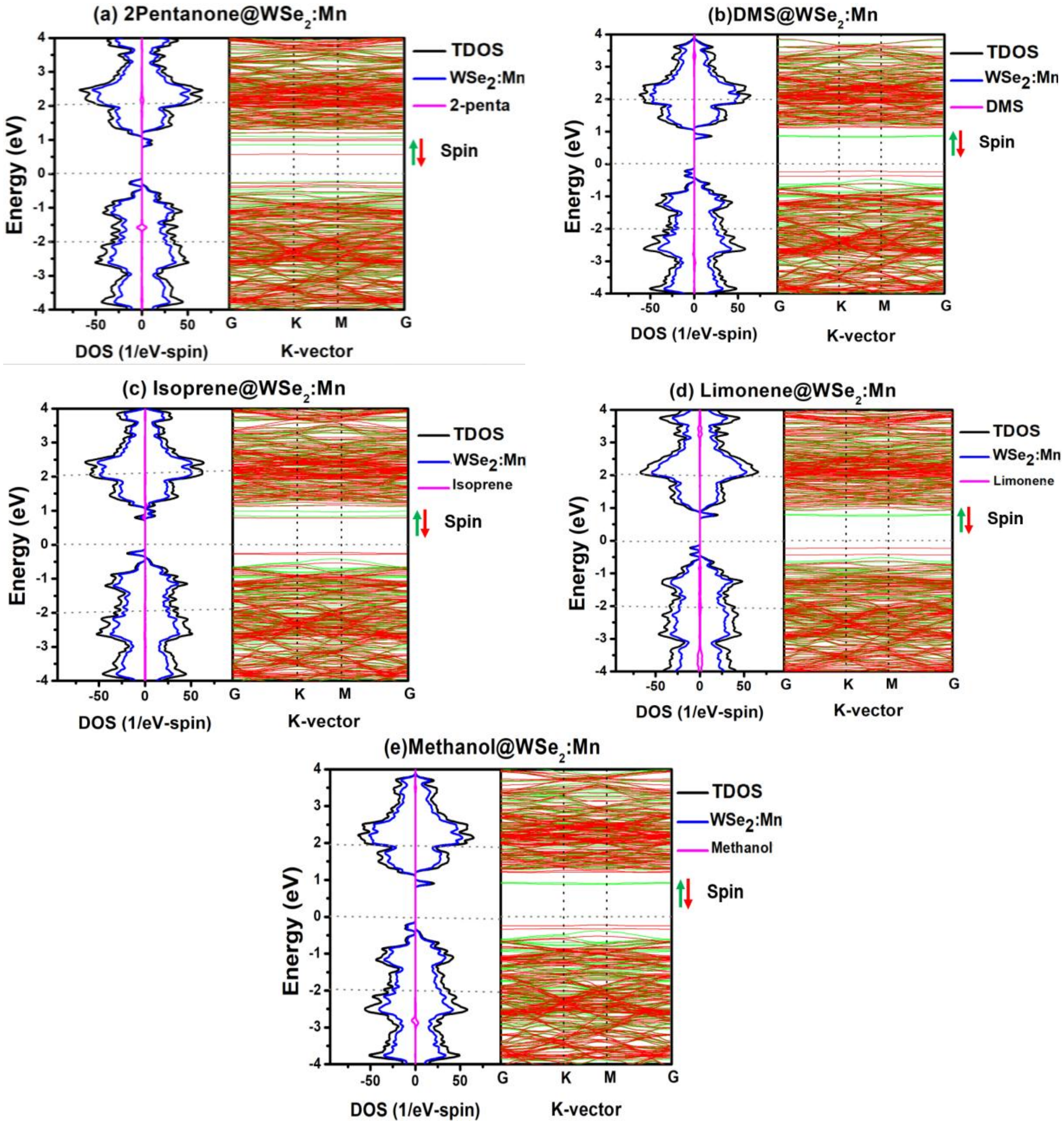
(a) 2Pentanone@WSe$_2$:Mn
TDOS
WSe$_2$:Mn
2-penta
Spin
Energy (eV)
DOS (1/eV-spin)
K-vector
G K M G
(b)DMS@WSe$_2$:Mn
TDOS
WSe$_2$:Mn
DMS
Spin
Energy (eV)
DOS (1/eV-spin)
K-vector
G K M G
(c) Isoprene@WSe$_2$:Mn
TDOS
WSe$_2$:Mn
Isoprene
Spin
Energy (eV)
DOS (1/eV-spin)
K-vector
G K M G
(d) Limonene@WSe$_2$:Mn
TDOS
WSe$_2$:Mn
Limonene
Spin
Energy (eV)
DOS (1/eV-spin)
K-vector
G K M G
(e)Methanol@WSe$_2$:Mn
TDOS
WSe$_2$:Mn
Methanol
Spin
Energy (eV)
DOS (1/eV-spin)
K-vector
G K M G

(B)

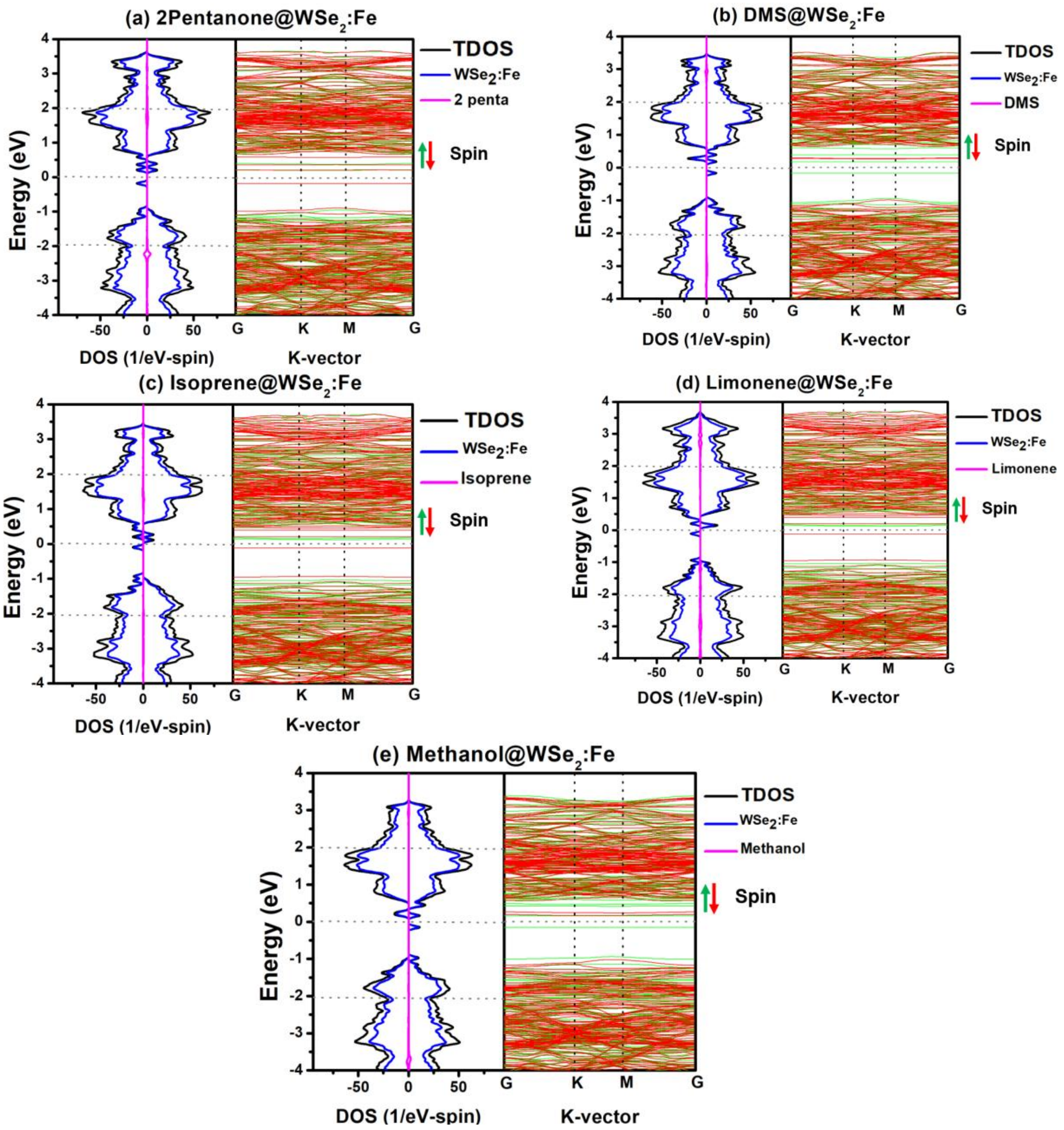

Figure S4: Band structures and PDOS/TDOS of five VOCs adsorbed on two samples: (A) WSe_2:Mn and (B) WSe_2:Fe.

(A)

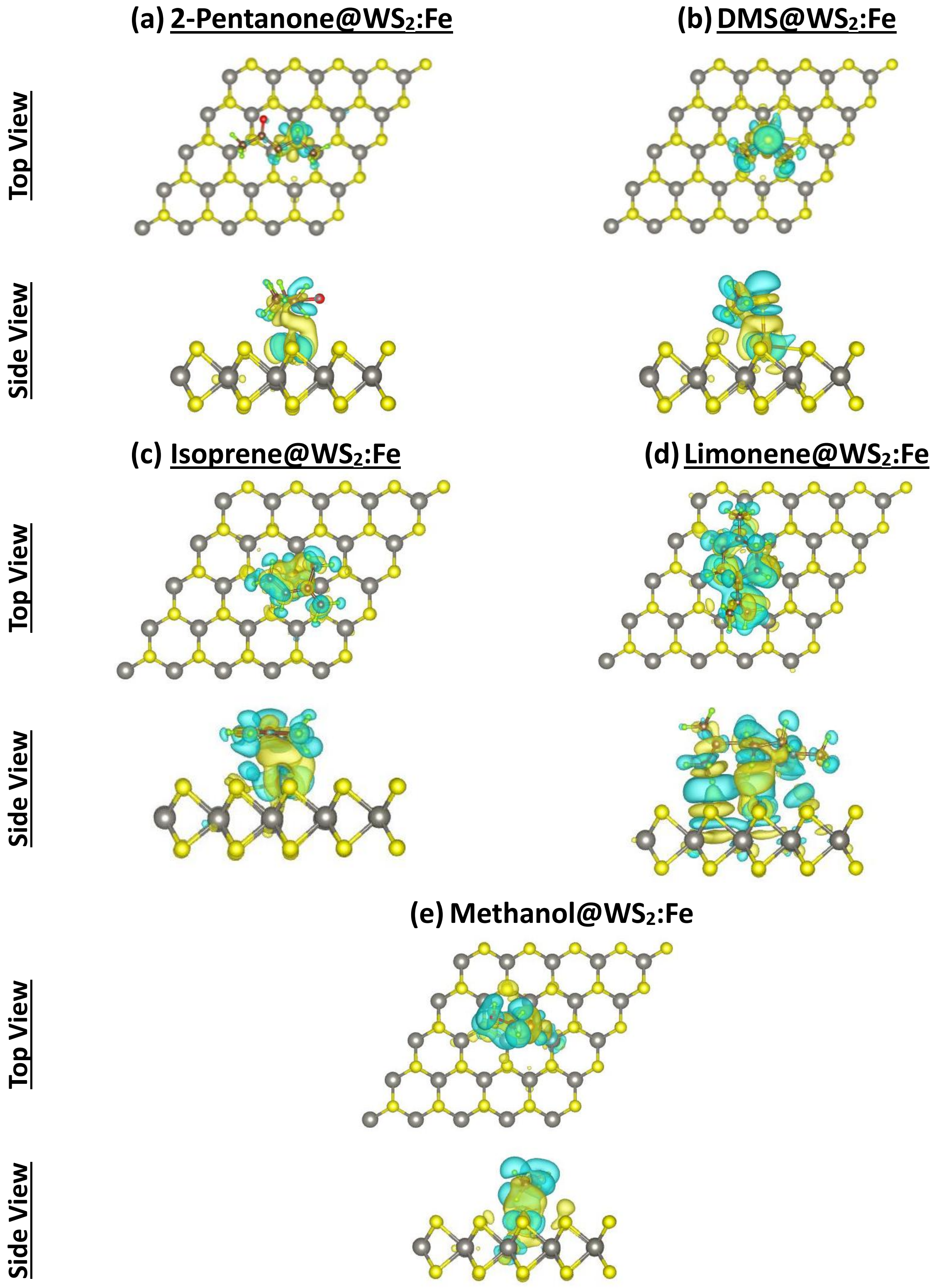
(a) 2-Pentanone@WS_2:Fe
(b) DMS@WS_2:Fe
Top View
Side View
(c) Isoprene@WS_2:Fe
(d) Limonene@WS_2:Fe
Top View
Side View
(e) Methanol@WS_2:Fe
Top View
Side View

(B)

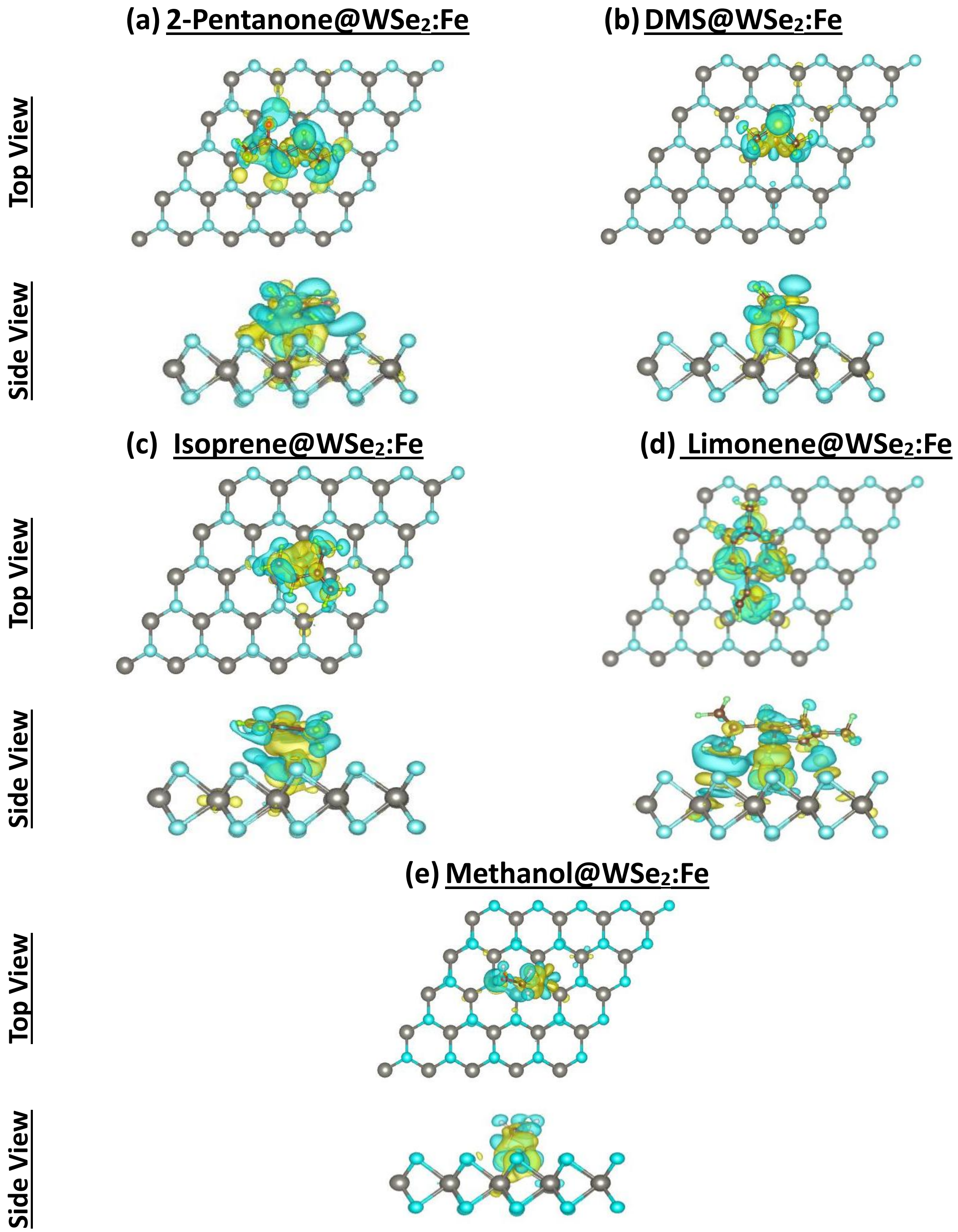

Figure S5: Charge density difference (CDD) due to the adsorptions of five VOC molecules on two samples: (A) WSe_2:Mn and (B) WSe_2:Fe.

Table S1: Results of adsorption energy, charge transfer and change of magnetization due to the adsorption of 9 molecules (5 VOCs and 4 air molecules) on four samples: (a) WS_2:Mn, (b) WS_2:Fe, (c) WSe_2:Mn, and (d) WSe_2:Fe.

	WS_2:Mn				WS_2:Fe				WSe_2:Mn				WS_2:Fe			
	E_{ads} (eV)	Δq (e)	ΔM (μ_B)	τ (s)	E_{ads} (eV)	Δq (e)	ΔM (μ_B)	τ (s)	E_{ads} (eV)	Δq (e)	ΔM (μ_B)	τ (s)	E_{ads} (eV)	Δq (e)	ΔM (μ_B)	τ (s)
**Methanol**	-1.32	0.46	0.018	2.10E+10	-1.79	0.38	0.008	1.88E+18	-1.62	0.42	0.066	2.5E+15	-1.84	0.32	0.04	1.32E+19
**DMS**	-1.85	0.68	0.029	1.94E+19	-2.18	0.61	8E-4	7.70E+24	-1.42	0.39	0.28	1.03E+12	-2.27	0.48	0.38	2.66E+26
**Limonene**	-2.31	0.89	0.181	1.17E+27	-2.45	0.44	0.13	2.73E+29	-2.19	0.79	0.11	1.09E+25	-1.98	0.37	0.02	3.19E+21
**Isoprene**	-2.19	0.44	0.02	1.09E+25	-2.64	0.32	0.27	5.11E+32	-2.49	0.61	0.11	1.30E+30	-2.10	0.40	0.08	3.69E+23
**2-pentanone**	-2.63	0.82	0.1	3.02E+32	-2.75	0.59	0.01	3.23E+34	-2.06	0.53	0.10	6.92E+22	-2.15	0.70	0.12	2.30E+24
**H_2O**	-0.36	0.03	0	1.23E-06	-0.19	0.003	0	1.70E-09	-0.21	0.01	0	3.56E-09	-0.18	0.01	0	1.11E-09
**N_2**	-0.22	0.01	0	4.50E-09	-0.12	0.014	0	1.07E-10	-0.16	0.00 6	0	5.08E-10	-0.20	0.01	0	2.32E-09
**CO_2**	-0.25	0.08	0	1.69E-08	-0.38	0.09	0	3.04E-06	-0.24	0.06	0	1.15E-08	-0.31	0.06	0	1.82E-07
**O_2**	-0.41	0.08	0	8.59E-06	-0.44	0.092	0	2.76E-05	-0.51	0.11	0	4.22E-04	-0.68	0.09	0	3.16E-0.9
